# Immigrant assimilation in health care utilisation in Spain

**DOI:** 10.1007/s10198-023-01622-6

**Published:** 2023-07-31

**Authors:** Zuleika Ferre, Patricia Triunfo, José-Ignacio Antón

**Affiliations:** 1 Department of Economics, Faculty of Social Sciences, University of the Republic (Uruguay), Montevideo, Uruguay; 2 Department of Applied Economics, Faculty of Economics and Business, University of Salamanca, Salamanca, Spain; 3 Instituto Universitario Gutiérrez Mellado, Universidad Nacional de Educación a Distancia, Madrid, Spain

**Keywords:** Migration, Health care, Assimilation, Spain, J15, I10

## Abstract

Abundant evidence has tracked the labour market and health assimilation of immigrants, including static analyses of differences in how foreign-born and native-born residents consume health care services. However, we know much less about how migrants’ patterns of healthcare usage evolve with time of residence, especially in countries providing universal or quasi-universal coverage. We investigate this process in Spain by combining all the available waves of the local health survey, which allows us to separately identify period, cohort, and assimilation effects. We find robust evidence of migrant assimilation in health care use, specifically in visits to general practitioners and emergency care and among foreign-born women. The differential effects of ageing on health care use between foreign-born and native-born populations contributes to the convergence of utilisation patterns in most health services after 15 years in Spain. Substantial heterogeneity by the time of arrival and by region of origin both suggest that studies modelling future welfare state finances would benefit from a more thorough assessment of migration.

**Supplementary Information:**

The online version contains supplementary material available at 10.1007/s10198-023-01622-6.

## Introduction

Migrants are common in societies of many different development levels Migration [IOM], [38]. Migrants’ overall success largely depends on their assimilation into the host society, including in how they access and use social services. Although immigration exerts overall beneficial effects on destination countries [37, Chapter 4], its impact on the sustainability of the welfare state is a subject of debate. These effects most likely depend on migrants’ characteristics, the specific features of welfare states, and the time horizon used to measure service use [11, 19, 29, 36, 52, 57]. In turn, as long as migration increases the ethnic heterogeneity of societies, it can also shape natives’ attitudes towards resource redistribution and the welfare state itself [4, 5, 20, 23].

This paper explores the patterns of migrants’ assimilation into health care in Spain. In particular, we evaluate how the length of residence affects migrants’ use of this service, considered the cornerstone of the Spanish welfare state. We also explore the extent of convergence in healthcare utilisation patterns between native and migrant populations, in terms of both assimilation and differential ageing processes.

To pursue these goals, we employ different econometric models to exploit four Spanish health surveys (conducted from 2010 to 2020). These surveys contain information on respondents’ time of residence in the country and include more than 80,000 natives and nearly 8,000 foreign-born individuals. This setup allows us to identify cohort, period, and assimilation effects and to carry out separate analysis by gender. Our findings suggest relevant migrant cohort effects in many services, i.e., foreign-born populations use some types of health care less than natives in their first years in the country. These results are consistent with both the healthy migrant effect and the persistence of information problems and language barriers.

Assimilation—in this context, the increase in health care utilisation with the number of years in the host country—is relevant for visits to general practitioners (GPs) and emergency care. Such an effect appears is specially robust for foreign-born females. We do not observe very large differences by region of origin or education, although the statistical power of our analyses substantially decreases when examining different groups of migrants separately. Similarly, the phenomenon is more pronounced for those migrants who arrived in the country during its economic boom from the mid-1990s to the Great Recession. We argue that this outcome might be due to health assimilation and limited socio-economic progress, given that the use of health care services in Spain is higher among individuals with high educational attainment.

Even if migrants use less health care upon arrival and their assimilation is quite limited, the age patterns of their utilisation are different than among locals. As a result, after 20 years in Spain, migrants tend to exhibit similar or higher levels of effective recourse to health services as the native population. Particularly, several arrival cohorts visit GPs more often and make more higher use of emergency care than the local population after 15 years in Spain.

Overall, our results suggest that the differences between comparable native and migrant populations in terms of health care utilisation are relatively minor when the latter arrive in the country. If anything, foreign-born individuals make lower use of health services on arrival, probably related to their health advantage. After some time, their behaviour becomes considerably similar. Nevertheless, we identify some services where health consumption seems higher among foreign-born population. We argue that this result might indicate a modest extra cost in terms of social spending due to migration. Further simulations assessing the costs and benefits of migration in Spain might profit from these findings.

To our knowledge, this study is the first to explore immigrant assimilation based on health care use in Spain. Whereas several papers provide evidence on how foreign-born populations exhibit better health than locals on arrival, how this gap tends to close with time of residence in the country [61, 62], and how foreign-born populations exhibit similar or lower health service utilisation (see, e.g., the surveys of [45, 55, 64], among many others), no previous work addresses this question linked to the dynamics of health care use.

Whereas the literature on immigrant health [9, 32, 35] and specially labour market assimilation [2, 14, 43] is abundant, few studies consider how patterns of immigrant health care use evolve with a time of residence in the host country. Studies focused on the U.S. indicate no difference in take-up rates of Medicaid means-tested benefits between natives and migrants, whose participation rises with the time spend in the country [16], as well as higher initial health expenditures among Latino migrants than locals, with certain evidence of convergence for Latino migrants who get American citizenship [80].

It is also worth summarising the findings from countries providing universal access to health care. Even though, overall, these studies suggest a lower use of non-emergency health care services by migrants (vs. natives) on arrival and a certain catch-up process, this literature is not unanimous. For instance, migrant males in Canada less frequently contact a doctor on arrival, but their levels of health care usage rise to match natives after 6–8 years in the country [47]. According to Wadsworth [82], the differences in healthcare use between migrants and natives in the United Kingdom and Germany are not large. In the U.K., foreigners make a little more use of GP services (but not hospitals) than natives. Their change in usage with time spent in the country does not follow a systematic pattern. In the case of Germany, if anything, migrants exhibit lower rates of the utilisation of healthcare (both general practitioners and hospital services), but their rates seem to converge with those of natives with the time of residence in the country. Finally, migrants’ usage of primary care emergency services in Norway exhibits substantial variation over groups and is higher than natives’ usage (perhaps at the expense of less effective access to other types of health care), and it decreases with the length of residence in the country [71].^1^

The remainder of our paper unfolds as follows. The second section provides some theoretical and institutional background for the analysis, while the third and fourth sections describe the database and methods employed in the analysis, respectively. Section 5 presents the empirical results and discusses their implications. Finally, in Section 6 we offer some concluding remarks and pathways from future research.

## Background

Like most developed countries, Spain provides virtually universal health care coverage to every resident in the country. National authorities extended this entitlement in 2000 to undocumented migrants, with the sole requirement being registration in a local population census (with no legal consequences). Nevertheless, in practical terms, information problems or fear of retaliation due to their irregular status, jointly with the hesitancy of some regional authorities (who are responsible for health care delivery) to provide foreigners without legal residence in Spain with health cards, could hamper actual access to the National Health System (NHS) among some segments of migrants.

In September 2012, in the middle of the Great Recession, the Spanish government restricted the access of undocumented foreigners to primary care (with the exceptions of minors, pregnant women, and anyone who needed emergency care). The reform still allowed the affected groups to purchase insurance for a monthly fee of 60 €  (individuals below 65) and 157 €  (individuals above 65). Some regional governments also promptly passed legislation to protect the affected populations and provide more beneficial insurance conditions, close to those before the reform. These restrictions might have resulted in less actual access to these services and worse health outcomes [41, 42].

The change in Spain’s central government in 2018, after a motion of no confidence, led to the restoration of the situation prior to the limitations on migrants’ health care access. In practice, similar obstacles apparently persist because of legal loopholes [81]. Bruquetas-Callejo and Perna [17] even argue that migrants’ entitlement to health care in Spain has been more of a political talking point than a subject with substantial differences between the two main political parties.

Previous literature on the theoretical reasons for expecting assimilation (or non-assimilation) in health care is scant. A first reason that increased use of health services may correspond with years living in the host country, particularly relevant in contexts without universal coverage, is the higher probability of improving health care access with longer residence in a region [9, 47, 80]. A second pertinent argument has to do with the existence of the “healthy migrant effect” and a process of negative assimilation in health, documented by recent studies focused on Spain [61, 62]. Similarly, during their first years in the country, migrants might face cultural and even linguistic barriers [59], whose relevance should decrease with their time spent in the country. A related argument refers to the role of information and knowledge on the functioning of health care systems, again likely to increase with time of residence in the host state [21]. This issue could have a negative effect on visits to GPs on arrival and a positive one on the use of emergency care [69, 70]. The existence of assimilation in this area is mostly an empirical question, since there are other factors that might act in the opposite direction. For instance, keeping in mind previous studies on the impact of income on health care demand, a non-linear but overall negative trend [10], assimilation of migrants in this domain might mitigate the increase in healthcare use—see, among many others, the survey of Antecol and Bedard [9].

## Data

Our analysis makes use of the National Health Survey (NHS), waves 2011–2012 and 2017 [75], and the European Health Interview Survey (EHIS), waves 2014 and 2020 [50], administered by the SSO. These databases are the first health surveys in Spain that include precise information on the timing of immigrants’ arrival in the country. The EHIS began much later than the NHS (whose first wave corresponds to 1987), but the EHIS is designed to be fully comparable to the NHS. Consequently, local authorities have discontinued the NHS, which they carried out roughly every three years until 2014. Both sources are representative of the resident population in the country aged at least 15 years old, at the regional level. Each wave includes approximately 24,000 households and follows a three-stage stratified sampling design, since it only selects one adult person from each household—households are randomly chosen from each census section—for an interview. Apart from basic socio-demographic characteristics, the questionnaires in both types of surveys cover detailed and comparable self-reported information on health status and health care utilisation. The main differences between the two sources is that the earlier questionnaire includes additional items on quality of life (e.g., information on social support) and additionally interviews a minor living each household. Hereafter, we refer to both questionnaires as national health surveys (NHSs).^2^

For the purpose of this investigation, we pool the samples of adults in the four waves mentioned above. We identify migrant status by looking at the country of birth rather than citizenship, because naturalization processes in Spain differ widely by state of origin (e.g., they are much shorter for people from some Latin American and Caribbean countries). Regarding health care use, we focus on the following items related to health services use: number of visits to general practitioners (GPs) in the last four weeks, number of visits to specialist doctors in the last four weeks, number of hospitalisations in the last 12 months, and number of times the person used emergency care in the last 12 months.^3^

The resulting sample, after dropping the observations with missing values on any of the variables included in the analysis (1.1% of cases), comprises 80,122 native and 7807 migrant adults. Using survey weights, the latter group represents 13.8% of the sample. Thanks to ad hoc agreements with the Spanish institutions responsible for granting access to the data (the SSO and the Ministry of Health, respectively), we are able to distinguish among foreign-born individuals from different regions of origin. The most relevant groups in demographic terms are migrants from Latin America and the Caribbean (43.4% of all the foreign-born adult population), those from European countries other than the European Union 15 (EU15) countries (19.7%), those from Africa (17.5%), and those from EU15 countries (13.6%). In contrast to other countries like Sweden or Germany, where refugees represent an important part of of the foreign population, the motivations for immigration to Spain are overwhelmingly economic, with the exception of EU15 migrants, who are mainly attracted by the benevolent climate and the lower cost of living than their countries of origin [76].^4^

We show the main summary statistics of the sample in Table 1. It includes both the variables used in our analyses of health care use assimilation and those considered for exploring the channels through which such a process takes place. In particular, we examine four health outcomes: self-perceived health (whether the respondent reports that his or her health has been very good over the past year), overweight status (based on self-reported height and weight) and mental health problems (a binary variable capturing whether the interviewed person reports having suffered from depression, anxiety or other mental conditions in the last 12 months).

**Table 1:** Descriptive statistics

	Means (standard deviations)	
	Natives	Migrants
No. of visits to a GP (last four weeks)	0.361	0.299
	(0.767)	(0.637)
No. of visits to a specialist (last four weeks)	0.137	0.100
	(0.546)	(0.445)
No. of hospitalisations (last year)	0.131	0.120
	(1.438)	(1.661)
No. of visits to emergency care (last year)	0.471	0.509
	(1.403)	(1.267)
Good or very good health (last year)	0.715	0.770
	(0.452)	(0.421)
Overweight (body mass index $\ge$ 25)	0.529	0.500
	(0.499)	(0.500)
Mental health problems (last year)	0.118	0.075
	(0.322)	(0.264)
Female	0.505	0.535
	(0.500)	(0.499)
Age	49.480	40.321
	(19.173)	(14.504)
Married	0.575	0.565
	(0.494)	(0.496)
Household size	2.923	3.291
	(1.235)	(1.554)
Low education	0.619	0.507
	(0.486)	(0.500)
Medium education	0.196	0.325
	(0.397)	(0.468)
High education	0.185	0.168
	(0.389)	(0.374)
Employed	0.452	0.533
	(0.498)	(0.499)
Unemployed	0.116	0.200
	(0.320)	(0.400)
Inactive	0.433	0.266
	(0.495)	(0.442)
Arrived before 1996		0.133
		(0.339)
Arrived between 1996 and 2007		0.623
		(0.485)
Arrived after 2007		0.244
		(0.430)
Less than 5 years since migration		0.131
		(0.338)
5–9 years since migration		0.245
		(0.430)
10–14 years since migration		0.294
		(0.456)
15 or more years since migration		0.329
		(0.470)
No. of observations	80,122	7807

*Notes*: The number of observations is lower in the case of overweight (75,359 natives and 7,497 migrants) and mental health problems (80,095 migrants and 7,807 natives). Observations are weighted using sampling weights

*Source*: Authors’ analysis from national health surveys

## Methods

In order to disentangle the effect of foreigners’ length of residence on their patterns of healthcare use, we adopt the empirical strategy utilized by Antecol and Bedard [8, 9] and Giuntella [30] for analysing health assimilation. Specifically, we estimate equations of the following form:

1 $$\begin{aligned} Y_{i} = {X_i}\beta + {A_i}\delta + {C_i}\gamma + {T_i}\pi + \varepsilon _{i}, \end{aligned}$$

where $Y_i$ denotes a health care variable relevant to person *i*, $X_i$ a vector of socio-demographic control variables (region fixed effects, degree of urbanisation, and a cubic of age, fully interacted with migrant status, education, marital and activity status and household size), $A_i$ a vector of dummy variables indicating how long an immigrant has lived in Spain (set equal to 0 for locals and excluding a category that serves as a reference), ${C_i}$ a vector of dummy variables identifying the arrival cohort (which takes the value 0 for natives), $T_i$ a vector of dummy variables capturing the survey year, and $\varepsilon _{i}$ a random disturbance.

We estimate equation 1 by Ordinary Least Squares (OLS) in our baseline analysis. We are not interested in prediction but in the marginal effects of the dummies due to cohort and assimilation variables. In this respect, OLS estimates are consistent under weaker assumptions than others obtained from count data models [7]. In any case, in Subsection 5.4, devoted to robustness checks, we present the results obtained using count data models.

Given the existence of relevant gender differences in health (related to genetics, social factors and lifestyle) and health care needs [12, 67, 72, 79], we estimate the equation of interest separately for men and women. As in the case of health status [9, 31], ethnicity might also play a role in health services utilisation. Whereas most of the population born in Spain is white, there is a considerable heterogeneity in the ethnic composition of migrant adults. This is due to the relevance of migration from Latin America, the Caribbean, and Africa. Previous research has identified relevant differences in the patterns of migrant health care use by state of origin [45]. For this reason, we additionally re-estimate our model comparing locals with migrants from the EU15, the rest of Europe, Latin America, and the Caribbean and Africa. Unfortunately, considering these groups separately largely reduces the available samples, which makes the estimates quite imprecise.

Pooling several cross-sections and both migrants and natives allows us to separately identify cohort, assimilation, and period effects. We consider three arrival cohorts based on Spain’s recent economic and social conditions: before 1996 (when massive immigration began), 1996–2007 (a period of strong economic growth before the financial crisis), and 2008–2020 (just after the start of the Great Recession). To study assimilation, we take into account four intervals of length of stay in Spain: 0–4, 5–9, 11–14 and 15 or more years. To identify the model, we omit the first category of time of residence. The coefficients due to arrival cohort indicate the differences in health care utilisation between migrants and natives at 0–4 years since migration. Those associated with the three dummies of the length of stay in the country (5–9, 10–14 and 15 or more years) capture the change in health care use for migrants with time spent in Spain. Combining the coefficients of the interaction between age and migrant status, the arrival cohort, and the time since arrival allow us to calculate how the migrant-native gap in healthcare use evolves over time. We can identify the period effect thanks to the inclusion of natives in the sample.

In principle, the coefficients of the binary variables due to migrant cohorts would indicate the gap in healthcare demand between locals and migrants on arrival at 0 years old. Therefore, to make the interpretation of the results easier, we centre age at 15 years old (the lowest age at which we can observe individuals in our database), so those parameters capture the difference between foreign-born individuals at 0–4 years since migration and native population at that age.^5^

We explicitly refrain from introducing variables controlling for health status in the left-hand side of equation, as those sort of variables are jointly determined with utilisation of healthcare services, and both are part of the process of immigrants’ assimilation to their host countries. To explore the potential role of these factors in shaping the use patterns of health services, we further estimate the role of immigrant assimilation in a set of health outcomes (self-reported health status, overweight status and prevalence of mental health problems).

The use of sparse cross-sectional data waves is the rule rather than the exception in this literature [9]. However, it is not free of problems. In particular, the lack of longitudinal data prevents us from tracking which migrants leave the country. Self-selection of return migration represents a potential threat to identification. For instance, if foreign-born populations who go back to their country of origin have worse health (and higher demand for health care) than stayers, the estimated coefficients would be inconsistent (downward biased). Regrettably, although the return migration became a very relevant phenomenon during the Great Recession and its aftermath [40, 44], there is little available evidence on the relationship between this phenomenon and health status for Spain. A survey of studies by Antecol and Bedard [9], focused on other countries, suggests mixed results, so the experience of other societies does not provide a clear guide here. If, as shown by Abramitzky et al [1] in the case of the labour market, negative self-selection of return migration were the norm, our estimates of assimilation would be a lower bound for the actual effect of the number of years spent by migrants in Spain.

## Results

### Main results

We display the main results of the econometric analysis in Table 2, which shows the estimates due to visits to GPs and specialists, and 3, which refers to hospital admissions and emergency care. Regarding the number of visits to GPs, we observe no difference in the frequency of use between locals and migrants on arrival in the case of males. Nevertheless, we observe that all the cohorts of foreign-born women make a less intense use of health services that their native counterparts. The length of residence in Spain only affect the utilisation of this service in the case of foreign-born men after 15 years (0.067 visits more than natives in the last four months). The frequency of use increases after 15 years in Spain by 0.189 visits among migrant women. The size of this assimilation effect is not negligible: it represents nearly half of the average number of visits to GPs. With regard to contacts with specialist doctors, assimilation is absent among both sexes. Nevertheless, it is worth mentioning that, on arrival, the female migrant cohort arriving between 2008 and 2020 exhibited a lower rate of utilisation of this service than natives. This effect (0.050 visits less) constitutes approximately one-third of the average number of contacts with specialists in Spain.

Table 3 shows the results for hospitalisations and emergency care. They reveal no difference between male migrants on arrival and locals for the former variable, but all the cohorts of foreign-born women exhibit substantially higher hospital admissions than comparable natives.^6^ Also, on arrival, two cohorts of migrant women exhibit a lower use of emergency services than female natives. Regarding assimilation effects, after 15 years in Spain, the number of emergency department visits by migrants exceeds that of the native population by 0.098 and 0.232 contacts, respectively. Those effects are relatively large (a quarter and more than one-third of the average number of visits, respectively)

**Table 2:** Age, immigrant arrival cohort and assimilation effects (OLS estimates) in visits to GP and specialist

	(I)	(II)	(III)	(IV)
	No. of visits to GP		No. of visits to specialist	
	Men	Women	Men	Women
Age effects and interactions				
Age	0.023***	$-$0.006***	0.014***	0.007***
	(0.002)	(0.001)	(0.001)	(0.001)
$\text {Age}^2 /100$	$-$0.034***	0.023***	$-$0.021***	$-$0.008***
	(0.003)	(0.002)	(0.002)	(0.001)
$\text {Age}^3$ /10,000	0.021***	$-$0.014***	0.009***	0.002***
	(0.002)	(0.001)	(0.001)	(0.001)
$\text {Age} \times \text {migrant}$	0.006	0.043***	$-$0.005	0.001
	(0.010)	(0.015)	(0.007)	(0.006)
$\text {Age}/100 \times \text {migrant}$	$-$0.018	$-$0.093***	0.007	$-$0.002
	(0.023)	(0.033)	(0.015)	(0.014)
$\text {Age}/{10,000} \times \text {migrant}$	0.012	0.057**	$-$0.004	0.002
	(0.016)	(0.023)	(0.010)	(0.009)
Immigrant arrival cohort				
Pre-1996	$-$0.037	$-$0.231***	0.002	$-$0.045
	(0.043)	(0.079)	(0.031)	(0.038)
1996–2007	$-$0.003	$-$0.187***	0.017	$-$0.049*
	(0.037)	(0.056)	(0.028)	(0.028)
2008–2020	0.011	$-$0.167***	$-$0.004	$-$0.050***
	(0.029)	(0.051)	(0.027)	(0.019)
Time of residence in Spain				
5–9 years	$-$0.016	0.064***	$-$0.008	$-$0.015
	(0.027)	(0.023)	(0.029)	(0.022)
10–14 years	0.015	0.112***	$-$0.008	0.037
	(0.030)	(0.029)	(0.026)	(0.031)
15 or more years	0.067**	0.189***	0.032	0.038
	(0.028)	(0.039)	(0.029)	(0.033)
Adjusted R^2^	0.050	0.033	0.031	0.033
No. of observations	40,936	46,993	40,936	46,993
Mean of dependent variable	0.293	0.410	0.107	0.156

*Notes*: *** significant at 1% level; ** significant at 5% level; * significant at 10% level. All specifications include an intercept, year and region fixed effects, degree of urbanisation, education, marital and activity status and household size. Observations are not weighted. Standard errors clustered at the cohort level in parentheses

*Source*: Authors’ analysis from national health surveys

**Table 3:** Age, immigrant arrival cohort and assimilation effects (OLS estimates) in hospital stays and visits to emergency care

	(I)	(II)	(III)	(IV)
	No. of hospitalisations		No. of visits to emergency care	
	Men	Women	Men	Women
Age effects and interactions				
Age	0.033***	0.023***	0.050***	0.028***
	(0.005)	(0.003)	(0.003)	(0.002)
$\text {Age}^2 /100$	$-$0.063***	$-$0.044***	$-$0.113***	$-$0.081***
	(0.009)	(0.006)	(0.007)	(0.004)
$\text {Age}^3$ /10,000	0.038***	0.027***	0.073***	0.057***
	(0.005)	(0.004)	(0.004)	(0.002)
$\text {Age} \times \text {migrant}$	$-$0.036	$-$0.019	$-$0.017	0.046**
	(0.037)	(0.011)	(0.018)	(0.021)
$\text {Age}/100 \times \text {migrant}$	0.097	0.026	0.038	$-$0.095**
	(0.100)	(0.030)	(0.040)	(0.044)
$\text {Age}/{10,000} \times \text {migrant}$	$-$0.070	$-$0.012	$-$0.031	0.058**
	(0.073)	(0.022)	(0.028)	(0.029)
Immigrant arrival cohort				
Pre-1996	0.193	0.230**	0.116	$-$0.336**
	(0.223)	(0.101)	(0.088)	(0.141)
1996–2007	0.232	0.185***	0.009	$-$0.209*
	(0.259)	(0.067)	(0.067)	(0.118)
2008–2020	0.260	0.144***	$-$0.024	$-$0.253**
	(0.284)	(0.046)	(0.072)	(0.098)
Time of residence in Spain				
5–9 years	$-$0.209	$-$0.021	0.073*	0.027
	(0.267)	(0.024)	(0.043)	(0.082)
10–14 years	$-$0.239	0.040	0.137***	0.115
	(0.278)	(0.073)	(0.051)	(0.094)
15 or more years	$-$0.276	0.022	0.098**	0.232**
	(0.307)	(0.049)	(0.044)	(0.106)
Adjusted R^2^	0.008	0.001	0.013	0.013
No. of observations	40,936	46,993	40,936	46,993
Mean of dependent variable	0.118	0.142	0.403	0.548

***Significant at 1% level

**Significant at 5% level

*Notes*: All specifications include an intercept, year and region fixed effects, degree of urbanisation, education, marital and activity status and household size. Observations are not weighted. Standard errors clustered at the cohort level in parentheses

*Source:* Authors’ analysis from national health surveys

### Heterogeneity of results

In this subsection, we explore how the results of assimilation to health care differ by country of origin, educational level and cohort of arrival. In the first two cases we use separate regressions, while we adopt the method proposed by Borjas [15] to explore which cohorts assimilate faster. Such an approach relies on a specification that comprises the interactions between the number of years in Spain and the cohort dummies.^7^

The analysis by place of birth is particularly interesting given the that the literature points to the existence of both racial health and healthcare disparities [66, 77, 84] as well as different cultural barriers as well (not only due to language but also due to the organisation and functioning of health care systems in the country of origin) [59, 70, 73, 78, 86].

Specifically, we look at foreign-born populations in the four most relevant groups of migrants: EU15, other European countries, Latin America, and the Caribbean and Africa.^8^ Because of sample size limitations, in many cases the estimated coefficients are not statistically different from zero, though they are not statistically different from our main results either. Therefore, we reproduce those results in the supplementary appendix (Table A1–A8) and we comment on the main salient findings here.

First, regarding EU15-born individuals (Table A1 and A2), most of estimated coefficient shows the same sign as in our main analysis, but they are quite imprecisely estimated and are not statistically significant. Second, the findings for other European migrants (Table A3 and A4) are roughly in line with the ones presented in Sect. 5.1. The most salient result is some evidence of assimilation in terms of visits to specialists, particularly for males. Third, in the case of foreign-born populations from Latin America and the Caribbean (Table A5 and A6), the time spent in Spain has a positive effect on women’s visits to GPs and specialists and also increases the number hospital admissions for males. Last, with regards to Africans (Table A7 and A8), the length of residence does not seem to affect healthcare use, (even though the estimated coefficients are not statistically different from those reported in the total sample), with the exception of visits to emergency departments by foreign-born men.

In terms of education, the assimilation process appears to be generally faster for the low-educated foreign-born than for the medium- and high-educated (Table A9–A12). The exception is emergency care, the use of which by migrant women increased by 0.445 contacts in the last year after 15 years in Spain.

Finally, we examine the differences in assimilation by arrival cohort (Table A13 and A14). First, our results suggest that the assimilation of male migrants in terms of GP visits is faster for the 1996–2007 arrival cohort than for the others. In the case of women, the rate of assimilation for this cohort only exceeds that of the pre-1996 cohort. Second, in terms of visits to specialists, for men the foreign-born population who arrived between 1996 and 2007 is assimilating faster than the earliest. For women, we cannot reject the lack of statistically significant differences among the three groups. Thirdly, no cohort experiences assimilation in terms of the number of hospitalisations. Finally, the difference between the cohorts in the number of visits to emergency departments is not statistically significant for men. For women, assimilation is faster for the central cohort than for the first one (migrants who arrived before 1996). We cannot rule out that there are no statistical differences between the speed of assimilation of the first and last cohorts of arrivals.

### Health care use after 15 years in Spain

As the number of years spent in Spain might impact the use of health care, and the utilisation patterns for these services might be different for migrants and natives, it is possible that the implications of migration for aggregate health spending vary over time. In this respect, our analysis suggests that on arrival, migrants are not an unusual burden in terms of expenditure. Their rates of utilisation are no higher than their native counterparts. The only exception is hospitalisations in the case of women. In this subsection, based on those estimates (i.e., net of the rest of characteristics considered in the analyses), we assess whether this impact changes after the foreign-born respondents have spent 15 years in Spain.

**Fig. 1:**
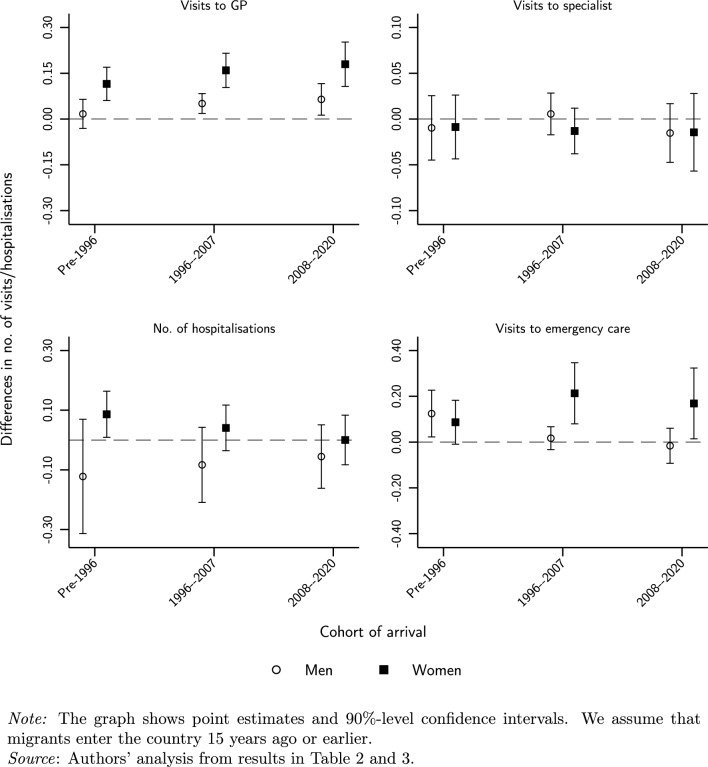
Differences in health care use between 35-year-old migrants after 15 years in Spain by arrival cohort

Figure 1 displays the differences in healthcare use between migrants and locals by type of service, sex, and arrival cohort. The results indicate that, after 15 years in Spain, foreign-born males do not use health care differently than natives, with the exception of GPs and the last two arrival cohorts whose rates of utilisation are higher than natives’ ones and the earliest cohort in terms of emergency department visits. Concerning migrant women, their number of visits to GPs is higher for natives, as the use of emergency care for migrants who arrived after 1996. It is also worth noting that the number of hospital admissions is slightly higher for migrant women of the earliest cohort who have lived in the country for 15 years or more than for natives.

In the appendix (Figure A1–A6), we present the results of separate analyses for four different groups of migrants by region of origin (EU15, other European countries, Latin America, and the Caribbean and Africa) and two educational segments. This exploration reveals substantial heterogeneity. For instance, some cohorts of Europeans exhibit lower rates of use of healthcare services than natives 15 years after arrival, while Latin American and Caribbean migrants contact GPs and emergency services substantially more than natives. Migrants with all levels of education tend to visit GPs at a higher frequency than their local counterparts. However, medium- and high-educated foreign-born individuals use more emergency services than natives and low-educated migrants.

These results illustrate that the impact of migration on health spending can vary depending on the time horizon considered and the origin of the foreign-born population. A quick back-of-the-envelope calculation taking into account only the four types of healthcare considered here, based on the total number of uses of health services [48, 49] and the cost estimated by some regional health authorities for each service [3, 58], exemplifies this point. After 15 years of living in Spain, migrants who arrived between 1996 and 2007 required roughly 5.6% higher health care spending than natives. Similarly, African individuals from the 2008–2020 arrival cohort show a lower level of consumption of these services—around 5.7% less—than locals.

Another interesting outcome that emerges from this picture is that the joint effect of assimilation and differential ageing tends to neutralise the initial differential use of health services by some foreign-born cohorts. In other words, we observe a process of convergence in terms of health care utilisation between migrants and locals, which in certain cases even results in higher rates of utilisation by migrants.

### Channels of assimilation

Although the assimilation described above is somewhat limited, it is interesting to dig deeper into the potential mechanisms driving this process, even in a speculative fashion. The first possible channel is health assimilation. In this respect, using a similar econometric specification, we explore the effect of time of residence in Spain on the probability of reporting good health, overweight status, or mental health problems (Table 4). Our results suggest that migrants’ health deteriorates over time in Spain relative to the local population. After 15 years in the country, the probability that the foreign-born population reports a good health status decreases by roughly 6 percentage points. The likelihood of migrant women being overweight increases by about 5 percentage points 15 years after arrival. Last, migrant males appear to declare a higher prevalence of mental health problems, by more than 4 percentage points.^9^

Our results suggest that assimilation in healthcare utilisation is greater for women than for men. These gender differences (i.e. stronger effects for migrant women) may partly reflect gender-specific job preferences and occupational segregation. Previous research suggests that women have stronger preferences for flexible jobs than men Goldin [33], which could explain the higher frequency of GP visits among female migrants than foreign-born males. The latter would face more difficulties in accessing these health services, which in Spain often have rather rigid practice opening hours, especially in the public sector, which dominates primary care.

The higher use of health care services among migrant women is not at odds with the findings of previous literature on Spanish immigration. Whereas the employment rates and earnings of foreign-born, working-age populations tend to increase with time of residence in Spain [6, 39], their occupational assimilation is incomplete [26, 63, 68]. Bearing in mind that health care use seems to decrease with occupational attainment in Spain [46], our results—indicating a higher number of visits to GPs by female migrants who have lived longer in Spain—would align with these labour market developments.

In a related argument, job quality, even leaving aside pay, is substantially worse for Spanish immigrants than for locals [22, 25, 27, 28]. Recent studies document that poor working conditions might have a detrimental effect on health similar to that of unemployment [18, 83]. Similarly, they suggest that the housing conditions of immigrants in Spain [56] are significantly worse than those of the native population.^10^ Such a disadvantage in dwelling quality can have far-reaching detrimental consequences on health [53].

A last potential channel has to do with acculturation. A relevant number of studies highlight the relevance of language and culture in migrants’ healthcare access [24, 54, 59, 70, 73, 78]. Our results are in line with this literature in that the foreign-born population segment experiencing the most intense assimilation processes are Latin Americans and Caribbeans, followed by Europeans, while the number of years in Spain does not seem to affect the rates of utilisation among Africans very much. Africans are arguably the most culturally distant migrant group from the Spanish locals.

**Table 4:** Immigrant arrival cohort and assimilation effects (OLS estimates) in health outcomes

	(I)	(II)	(III)	(IV)	(V)	(VI)
	Good health		Overweight		Mental health problems	
	Men	Women	Men	Women	Men	Women
Immigrant arrival cohort						
Pre-1996	$-$0.056	0.106***	0.000	$-$0.130***	0.035*	$-$0.022
	(0.036)	(0.037)	(0.052)	(0.045)	(0.019)	(0.033)
1996–2007	$-$0.034	0.083***	0.019	$-$0.062*	$-$0.004	$-$0.023
	(0.030)	(0.031)	(0.045)	(0.036)	(0.015)	(0.019)
2008–2020	$-$0.022	0.113***	$-$0.018	$-$0.031	$-$0.002	$-$0.061***
	(0.031)	(0.025)	(0.043)	(0.038)	(0.013)	(0.018)
Time of residence in Spain						
5–9 years	$-$0.048**	$-$0.030*	0.003	0.052**	0.027***	$-$0.008
	(0.023)	(0.017)	(0.032)	(0.026)	(0.008)	(0.018)
10–14 years	$-$0.060***	$-$0.050**	0.024	0.070***	0.047***	$-$0.009
	(0.021)	(0.022)	(0.038)	(0.025)	(0.010)	(0.015)
15 or more years	$-$0.062**	$-$0.063***	0.027	0.052**	0.044***	0.013
	(0.026)	(0.023)	(0.038)	(0.021)	(0.012)	(0.026)
Adjusted R^2^	0.152	0.182	0.098	0.129	0.042	0.057
No. of observations	40,936	46,993	39,508	43,348	40,926	46,976
Mean of dependent variable	0.765	0.681	0.605	0.445	0.073	0.150

*Notes*: *** significant at 1% level; ** significant at 5% level; * significant at 10% level. All specifications include an intercept, year and region fixed effects and controls for age (introduced through a third-degree polynomial fully interacted with migrant status), degree of urbanisation, education, and marital and activity status and household size. Observations are not weighted. Standard errors clustered at the cohort level in parentheses

*Source*: Authors’ analysis from national health surveys

### Robustness checks

In this section, we comment on the results of several robustness checks that test the sensitivity of our results to different methodological choices. First, we assess the stability of our results when including a different categorisation of the variable of interest (years in the host country). In particular, we consider a new set of dummy variables (0–2, 3–7, 8–12 and 13 or more years in Spain) and a linear specification of the length of stay in Spain (Table A17–A20). Both sets of results are very similar to our baseline ones.

Second, we verify that our main findings do not change when controlling for regional linear time trends (Table A21 and A22). Third, we re-estimate all our models using a Poisson regression model (Table A23 and A24 in the online appendix). Like OLS, this approach yields consistent estimates without requiring any further function of the error term [13, 85].^11^ Reassuringly, the results are pretty similar to our baseline estimations.^12^

Our fourth robustness exercise consists of performing our estimations while including only those individuals who solely have public health insurance, as a way of isolating our results from the eventual distortions due to the different normative changes in the last decade (Table A25 and A26). Again, our findings mostly hold, even though the degree of precision diminishes because of the smaller sample.^13^ The last sensitivity check explores whether our results vary when we limit our analysis to individuals aged less than 65 years old (Table A27 and A28). This methodological choice, which might help to ameliorate the eventual bias due to return migration, does not seem to have any influence on our results beyond reducing the sample in a non-trivial way. In these two sensitivity exercises, the estimated coefficient for assimilation in GP visits for migrant men becomes insignificant. This change reflects the reduction in precision due to the smaller sample size, as the point estimate is not statistically different from the baseline analysis.

## Conclusion

Immigration’s relationship with the welfare state demands substantial attention from both policy makers and society as a whole. The access and use of health care by foreign-born populations represents a matter of relevance because of their effect on public finances, but also in terms of ensuring an adequate integration of immigrants in the host country. The results of the assessment of both issues might differ on migrants’ arrival and after a longer term of residence.

This research contributes to the literature by providing detailed evidence on the process of assimilation in health care utilisation by immigrants in Spain. Our findings suggest, first, that some population segments of foreign-born populations, on arrival, use part of these services less than comparable natives, which is compatible with the healthy migrant effect. Furthermore, such use increases with time living in Spain. In particular, this phenomenon characterises visits to GPs and emergency care. For women, the frequency of use of these services is lower than for nationals but increases significantly with the length of stay in the country.

Although the evidence of assimilation does not apply to all types of health care, it is far not negligible either: public health spending represented more than 50% of total expenditures in these areas in 2019 [49, 60]. As a result of this limited assimilation and the different impact of age on the rates of health care utilisation, the patterns of consumption for these services by migrants converge with the natives’ figures. For a number of migrants cohorts and certain types of health care, the foreign-born individuals’ rates even exceed those of the native population. Note that the impact of the time spent in the host country (assimilation) and the differential effect of age on the variable of interest represent distinct phenomena. We can separate them in our analysis, thanks to the use of several waves of the database and the consideration of both natives and migrants in the main specification.

Our results suggest that even if the gap between migrants and natives in health care use is narrow, assimilation plays a significant role. The existence of substantial heterogeneity by migrant group and time passed since arrival might influence the estimates of migrants’ impact on public finances. As a consequence, we believe that researchers aiming to judge welfare state sustainability and those that cover migration could benefit from a more detailed modelling of the patterns of access to social services by foreign-born populations.

### Supplementary Information

Below is the link to the electronic supplementary material.

Supplementary file1 (PDF 364 KB)

## Footnotes

1 1Schober and Zocher [65], who only consider asylum-seekers (a very specific subset of the foreign-born population) and evaluate them five years after arrival, find that the effect of time of residence in Austria on health expenditures is negative for refugees and not clear for economic migrants: the evolution of the results for stayers is different than for the rest of the foreign-born population in this group.

2 The Spanish Statistical Office [74], which is responsible for both surveys, has been conducting them since 2014 in such a way that they are fully comparable, with the explicit aim of integrating the EHIS into the national statistical system. The EU has mandated its Member States to carry out this survey since 2011 and, since 2014, the Spanish government has ensured that the design of the EHIS in Spain allows using this database to monitor several dimensions of health and health care, alongside the already existing national health surveys carried out approximately every four or five years.

3 We rely on self-reported information, which is a not insignificant limitation, as the inability to accurately recall episodes of health care use may lead to loss of information. In particular, this circumstance could introduce some measurement error in the left-hand variable (in the case of classical measurement error, our estimates will be less precise).

4 There is a small share of migrants from the Southern Cone of Latin America who immigrated to Spain for political reasons in the eighties [51].

5 The inclusion of region-of-origin in this specification does not alter our results, since the composition of immigrant flows by place of birth does not change very much over time. Such dummies would capture region-of-origin differences in health care on arrival. This would make their comparison with the average estimated assimilation effects less intuitive and immediate. Furthermore, we estimate separate models by region of origin in the heterogeneity analysis of the results.

6 This result probably reflects the fact that migrants’ fertility rates on arrival are higher than natives’ rates (Spain has one of the lowest native fertility rates in the world), correlated with the ones observed in their country of origin and decreasing with time spent in Spain [34].

7 Although we favour non-linear specifications over this parametric approach, we adopt the latter because of sample size limitations.

8 The rest of the immigrants represent less than 6% and less than 1% of the foreign-born and total populations, respectively. They include individuals from very different origins, like the United States or Asia. For these reasons, our analysis does not consider them.

9 Our results are largely consistent with those reported by previous work for Spain that only exploited a single wave of the NHS [61, 62]. We also look at the impact of time spent in Spain on obesity, alcohol consumption, smoking and physical activity (Table A15 and A16), without finding any additional evidence of negative assimilation.

10 For example, the proportion of the foreign-born population living in overcrowded households (1.6% of the native population vs. 7.5% of the foreign-born population) or in substandard housing (3.9% vs. 7.7%) is much higher than for the locals [56].

11 This model is not efficient in cases of over-dispersion. Nevertheless, other alternatives like the negative binomial regression model, which is more efficient if the error term follows the assumed functional form, result in biased and inconsistent estimates in case of the violation of such properties.

12 One should bear in mind that, roughly, the coefficients of the Poisson regression model indicate the percentage change in the left-hand-side variable associated with a one-unit change in the right-hand-side variable.

13 Although insurance is an endogenous variable, the share of the population with only this coverage, almost all migrants and more than 60% of locals, is representative enough to be interesting on its own.

## References

[1] Abramitzky R, Boustan L, Eriksson K (2020). Do immigrants assimilate more slowly today than in the past?. American Economic Review: Insights.

[2] Abramitzky R, Boustan LP, Eriksson K (2014). A nation of immigrants: assimilation and economic outcomes in the Age of Mass Migration. Journal of Political Economy.

[3] Acuerdo de 5 de febrero de 2019, del Consejo de Administración del Ente Público Osakidetza-Servicio vasco de salud, por el que se aprueban las tarifas por prestación de servicios sanitarios y docentes a terceros obligados al pago durante el ejercicio 2019, Boletín Oficial del País Vasco, 46, 6th March 2019 (2019). https://www.euskadi.eus/bopv2/datos/2019/03/1901213a.pdf

[4] Alesina A, Harnoss J, Rapoport H (2021). Immigration and the future of the Welfare State in Europe. The Annals of the American Academy of Political and Social Science.

[5] Alesina A, Murard E, Rapoport H (2021). Immigration and preferences for redistribution in Europe. Journal of Economic Geography.

[6] Amuedo-Dorantes C, de la Rica S (2007). Labour market assimilation of recent immigrants in Spain. British Journal of Industrial Relations.

[7] Angrist, J.D., Pischke, J.-S.: Mostly harmless Econometrics: an empiricist’s companion. Princeton University Press (2008)

[8] Antecol H, Bedard K (2006). Unhealthy assimilation: why do immigrants converge to American health status levels?. Demography.

[9] Antecol, H., Bedard, K.: Immigrants and immigrant health. In B. R. Chiswick & P. W. Miller (Eds.), Handbook of the Economics of International Migration (pp. 271-314, Vol. 1A) (2015). North Holland. 10.1016/B978-0-444-53764-5.00006-2

[10] Antón J-I, Muñoz de Bustillo R (2010). Health care utilisation and immigration in Spain. European Journal of Health Economics.

[11] Barrett A, McCarthy Y (2008). Immigrants and welfare programmes: exploring the interactions between immigrant characteristics, immigrant welfare dependence, and welfare policy. Oxford Review of Economic Policy.

[12] Bird CE, Rieker PP (1999). Gender matters: an integrated model for understanding men’s and women’s health. Social Science & Medicine.

[13] Blackburn ML (2014). The relative performance of Poisson and negative binomial regression estimators. Oxford Bulletin of Economics and Statistics.

[14] Bodvarsson, Ö.B., Van der Berg, H.: The Economics of Immigration: theory and policy. Springer (2013)

[15] Borjas GJ (2015). The slowdown in the economic assimilation of immigrants: aging and cohort effects revisited again. Journal of Human Capital.

[16] Borjas GJ, Hilton L (1996). Immigration and the welfare state: immigrant participation in means-tested entitlement programs. Quarterly Journal of Economics.

[17] Bruquetas-Callejo M, Perna R (2020). Migration and healthcare reforms in Spain: symbolic politics, converging outputs, oppositions from the field. South European Society and Politics.

[18] Chandola T, Zhang N (2017). Re-employment, job quality, health and allostatic load biomarkers: prospective evidence from the UK Household Longitudinal Study. International Journal of Epidemiology.

[19] Christl M, Bélanger A, Conte A, Mazza J, Narazani E (2022). Projecting the fiscal impact of immigration in the European Union. Fiscal Studies.

[20] Dahlberg M, Edmark K, Lundqvist H (2012). Ethnic diversity and preferences for redistribution. Journal of Political Economy.

[21] Devillanova C (2008). Social networks, information and health care utilization: evidence from undocumented immigrants in Milan. Journal of Health Economics.

[22] Díaz-Serrano L (2013). Immigrants, natives and job quality: evidence from Spain. International Journal of Manpower.

[23] Facchini G, Mayda AM, Murard E, Freeman GP, Mirilovic N (2016). Does immigration affect preferences for redistribution? evidence across countries. Handbook on migration and social policy.

[24] Fassaert T, Hesselink AE, Verhoeff AP (2009). Acculturation and use of health care services by Turkish and Moroccan migrants: a cross-sectional population-based study. BMC Public Health.

[25] Fernández C, Ortega C (2007). Labor market assimilation of immigrants in Spain: employment at the expense of bad job-matches?. Spanish Economic Review.

[26] Fernández-Macíías E, Grande R, del Rey Poveda A, Antón J-I (2015). Employment and occupational mobility among recently arrived immigrants: the Spanish case 1997–2007. Population Research and Policy Review.

[27] Gálvez-Iniesta I (2022). The cyclicality of immigrant wages and labour market flows: evidence from Spain. Economics.

[28] Gamero Burón C (2010). Satisfacción laboral de los asalariados immigrantes. Revista de Economía Aplicada.

[29] Giulietti C (2014). The welfare magnet hypothesis and the welfare take-up of migrants. IZA World of Labor.

[30] Giuntella O (2016). Assimilation and health: evidence from linked birth records of second- and third-generation Hispanics. Demography.

[31] Giuntella, O., Lonsky, J.: Migration and health. In K. F. Zimmermann (Ed.), Handbook of Labor, Human Resources and Population Economics (pp. 1-15). Springer (2022). 10.1007/978-3-319-57365-6_98-1

[32] Giuntella O, Stella L (2016). The acceleration of immigrant unhealthy assimilation. Health Economics.

[33] Goldin, C.: Career and family: women’s century-long journey toward equity. Princeton University Press (2021)

[34] Grande R, Del Rey A (2017). The fertility of Latin American and Caribbean women in Spain: adaptation, maintenance or interruption?. Papeles de Población.

[35] Hamilton E, Berger Cardoso J, Hummer RA, Padilla YC (2011). Assimilation and emerging health disparities among new generations of U.S. children. Demographic Research.

[36] Hansen MF, Schultz-Nielsen ML, Tranæs T (2017). The fiscal impact of immigration to welfare states of the Scandinavian type. Journal of Population Economics.

[37] International Monetary Fund. World Economic Outlook, April 2020: the Great Lockdown. IMF (2020)

[38] International Organization for Migration. World Migration Report 2020. IOM (2019)

[39] Izquierdo M, Jimeno JF, Aitor L (2016). Spain: from massive immigration to vast emigration?. IZA Journal of Migration.

[40] Izquierdo M, Jimeno JF, Rojas JA (2010). On the aggregate effects of immigration in Spain. SERIEs-Journal of the Spanish Economic Association.

[41] Jiménez-Rubio D, Vall-Castelló J (2020). Limiting health-care access to undocumented immigrants: a wise option?. Health Economics.

[42] Juanmarti Mestres A, López Casasnovas G, Vall Castelló J (2021). The deadly effects of losing health insurance. European Economic Review.

[43] Lalonde, R. J., Topel, R. H. Economic impact of international migration and economic performance of migrants. In M. R. Rosenzweig & O. Stark (Eds.), Handbook of Population and Family Economics (pp. 799-850, Vol. 1B). North Holland (1997). 10.1016/S1574-003X(97)80006-2

[44] Larramona G (2013). Out-migration of immigrants in Spain. Population.

[45] Llop-Gironés A, Vargas Lorenzo I, Garcia-Subirats I, Aller M-B, Vázquez Navarrete ML (2014). Acceso a los servicios de salud de la población inmigrante en España. Revista Española de Salud Pública.

[46] Lostao L, Regidor E, Gimeno D, Netuveli G, Blane D (2011). Socioeconomic patterns in health services use in Great Britain and Spain before and after the health system reforms of the 1990s. Health & Place.

[47] McDonald JT, Kennedy S (2004). Insights into the “healthy immigrant effect”: health status and health service use of immigrants to Canada. Social Science & Medicine.

[48] Ministerio de Sanidad. Evolución de la estancia media hospitalaria en los hospitales de agudos del Sistema Nacional de Salud: años 2010-2019 (2021). https://www.sanidad.gob.es/estadEstudios/estadisticas/docs/Informe_EMH.pdf

[49] Ministerio de Sanidad. Informe anual del Sistema Nacional de Salud 2020-2021 (2022). https://www.sanidad.gob.es/estadEstudios/estadisticas/sisInfSanSNS/tablasEstadisticas/InfAnualSNS2020_21/INFORME_ANUAL_2020_21.pdf

[50] Ministry of Health: National Health Survey [Data set]. Ministry of Health, Madrid (2022)

[51] Muñoz de Bustillo R, Antón J-I (2010). De la España que emigra a la España que acoge: contexto, dimensión y características de la inmigración latinoamericana en España. América Latina Hoy.

[52] Nannestad P (2007). Immigration and welfare states: a survey of 15 years of research. European Journal of Political Economy.

[53] Navarro C, Ayala L, Labeaga JM (2010). Housing deprivation and health status: evidence from Spain. Empirical Economics.

[54] Ndumbi, P., del Romero, J., Pulido, F., Velasco Arribas, M., Fernando, D., Blanco Ramos, J. R., García de Olalla, P., Ocaña, I., Belda-Ibañez, J., del Amo, J., Álvarez-del Arcom, D., & The AMASE Research Group: Barriers to health care services for migrants living with HIV in Spain. European Journal of Public Health **28**(3), 451–457 (2018). 10.1093/eurpub/ckx225 10.1093/eurpub/ckx225 29325097

[55] Norredam M, Nielsen SS, Krasnik A (2009). Migrants’ utilization of somatic healthcare services in Europe-a systematic review. European Journal of Public Health.

[56] OECD & European Union. Indicators of immigrant integration 2015 (2015): settling in. 10.1787/9789264234024-en

[57] Organisation for the Co-operation and Economic Development: International Migration Outlook 2021. OECD Publishing (2021). 10.1787/29f23e9d-en

[58] Osakidetza-Servicio Vasco de Salud. (2023, March 9). Coste efectivo de los servicios de salud. Osakidetza-Servicio Vasco de Salud, Departamento de Salud. https://www.osakidetza.euskadi.eus/transparencia-buen-gobierno/-/costeefectivo-servicios-de-salud/

[59] Pena Díaz, C. (2016). Linguistic and pragmatic barriers in immigrant health care in Spain: the need for interlinguistic & intercultural mediators. Entreculturas. Revista de traducción y comunicación intercultural, (7-8), 625-634. 10.24310/Entreculturasertci.vi7-8.11358

[60] Resolución STL/353/2013 de 13 de febrero, sobre la revisión de precios públicos correspondientes a los servicios sanitarios que presta el Instituto Catalán de la Salud, Diari Oficial de la Generalitat de Catalunya, 6429, 1st March 2013 (2013). https://dogc.gencat.cat/es/document-del-dogc/?documentId=629759

[61] Rivera B, Casal B, Currais L (2013). Healthy immigrant effect: trayectoria de salud de la población inmigrante a partir de la ENSE 2011–2012. Estudios de Economía Aplicada.

[62] Rivera B, Casal B, Currais L (2015). Length of stay and mental health of the immigrant population in Spain: evidence of the healthy immigrant effect. Applied Economics.

[63] Rodríguez-Planas N, Nollenberger N (2016). Labor market integration of new immigrants in Spain. IZA Journal of Labor Policy.

[64] Sarría-Santamera, A., Hijas-Gómez, A. I., Carmona, R., Gimeno-Feliú, L. A.: A systematic review of the use of health services by immigrants and native populations. Public Health Reviews, **37**, Article 28 (2016). 10.1186/s40985-016-0042-3 10.1186/s40985-016-0042-3 PMC5810113 29450069

[65] Schober, T., Zocher, K.: Health-care utilization of refugees: evidence from Austria. International Migration Review. Advance online publication (2022). 10.1177/01979183211061091

[66] Semiha D, Koopmans G, Birnie E, Foets M, Bonsel G (2009). Ethnic background and differences in health care use: a national cross-sectional study of native Dutch and immigrant elderly in the Netherlands. International Journal for Equity in Health.

[67] Short SE, Yang YC, Jenkins TM (2013). Sex, gender, genetics, and health. American Journal of Public Health.

[68] Simón H, Ramos R, Sanromá E (2014). Immigrant occupational mobility: longitudinal evidence from Spain. European Journal of Population.

[69] Småland Goth UG, Berg JE (2010). Migrant participation in Norwegian health care. a qualitative study using key informants. European Journal of General Practice.

[70] Småland Goth UG, Berg JE, Akman H (2010). The intercultural challenges of general practitioners in Norway with migrant patients. International Journal of Migration, Health and Social Care.

[71] Småland Goth UG, Godager G (2012). Use of primary care emergency services in Norway: impact of birth country and duration of residence. Nordic Journal of Health Economics.

[72] Socías ME, Koehoorn M, Shoveller J (2016). Gender inequalities in access to health care among adults living in British Columbia. Canada. Women’s Health Issues.

[73] Sorensen J, Norredam M, Suurmond J, Carter-Pokras O, Garcia-Ramirez M, Krasnik A (2019). Need for ensuring cultural competence in medical programmes of European universities. BMC Medical Education.

[74] Spanish Statistical Office. Encuesta Europea de Salud en España 2020 (2020). metodología. https://www.ine.es/metodologia/t15/t153042020.pdf

[75] Spanish Statistical Office: European Survey of Health in Spain [Data set]. SSO, Madrid (2022)

[76] Spanish Statistical Office: National Immigrant Survey [Data set]. Spanish Statistical Office, Madrid (2022)

[77] Stronks, K., Snijder, M.B., Peters, R.J.G., Prins, M., Schene, A.H., Zwinderman, A.H.: Unravelling the impact of ethnicity on health in europe: the HELIUS study. BMC Public Health **13**(1), (2013). 10.1186/1471-2458-13-402 10.1186/1471-2458-13-402 PMC3646682 23621920

[78] Thomas F, Thomas F (2016). Cultural competence in migrant healthcare. Handbook of Migration and Health.

[79] van Wijk CMG, van Vliet KP, Kolk AM (1996). Gender perspectives and quality of care: towards appropriate and adequate health care for women. Social Science & Medicine.

[80] Vargas Bustamante A, Chen J (2011). Health expenditure ddynamics and years of U.S. residence: analyzing spending disparities among Latinos by citizenship/nativity status. Health Services Research.

[81] Villarreal, A.: Un año de la “sanidad universal” de Pedro Sánchez: “Es incluso peor que la del PP”. El Confidencial (2019). 10.1016/j.euroecorev.2020.103608

[82] Wadsworth J (2013). Mustn’t grumble: immigration, health and health service use in the UK and germany. Fiscal Studies.

[83] Wang S, Kamerāde D, Burchell B, Coutts A, Balderson SU (2022). What matters more for employees’ mental health: job quality or job quantity?. Cambridge Journal of Economics.

[84] Weinick RM, Zuvekas SH, Cohen JW (2000). Racial and ethnic differences in access to and use of health care services, 1977 to 1996. Medical Care Research and Review.

[85] Wooldridge, J. M.: Econometric analysis of cross section and panel data (2nd ed.). The MIT Press (2010)

[86] Zamora ER, Kaul S, Kirchhoff AC, Gwilliam V, Jimenez OA, Morreall DK, Montenegro RE, Kinney AY, Fluchel MN (2016). The impact of language barriers and immigration status on the care experience for Spanish-speaking caregivers of patients with pediatric cancer. Pediatric Blood & Cancer.

