# Supplementary material for: Immigrant assimilation in health care utilisation in Spain
Source: Eur J Health Econ. 2023 Jul 31;25(4):701–15. doi: 10.1007/s10198-023-01622-6 (PMC11136863; doi:10.1007/s10198-023-01622-6)
Supplement: Supplementary file 1 — Supplementary file1 (PDF 364 KB) [file 10198_2023_1622_MOESM1_ESM.pdf]

Supplementary appendix

Table A1. Age, immigrant arrival cohort and assimilation effects (OLS estimates) in visits to GP and specialist (foreign-born population from EU15 countries)

	(I)	(II)	(III)	(IV)
	No. of visits to GP		No. of visits to specialist	
	Men	Women	Men	Women
Age effects and interactions				
Age	0.024***	–0.007***	0.015***	0.007***
	(0.000)	(0.000)	(0.000)	(0.000)
$Age^2/100$	–0.036***	0.024***	–0.022***	–0.007***
	(0.001)	(0.001)	(0.001)	(0.001)
Age^3 /10000	0.022***	–0.015***	0.010***	0.001***
	(0.000)	(0.000)	(0.000)	(0.000)
Age × migrant	0.060**	0.050	0.002	–0.035*
	(0.027)	(0.034)	(0.013)	(0.019)
Age/100 × migrant	–0.133**	–0.117	–0.011	0.068*
	(0.059)	(0.075)	(0.024)	(0.038)
Age/10,000 × migrant	0.086**	0.078	0.008	–0.042*
	(0.040)	(0.052)	(0.015)	(0.023)
Immigrant arrival cohort				
Pre–1996	–0.115	–0.131	0.070	0.114
	(0.119)	(0.203)	(0.152)	(0.128)
1996–2007	–0.182*	–0.305*	0.077	0.075
	(0.100)	(0.181)	(0.146)	(0.112)
2008–2020	–0.237***	–0.180	0.011	0.104
	(0.085)	(0.135)	(0.096)	(0.111)
Time of residence in Spain				
5–9 years	–0.049	0.047	–0.043	–0.024
	(0.061)	(0.091)	(0.067)	(0.047)
10–14 years	–0.032	0.223	–0.085	0.046
	(0.061)	(0.165)	(0.069)	(0.062)
15 or more years	–0.026	0.084	–0.021	0.062
	(0.070)	(0.140)	(0.084)	(0.067)
Adjusted R^2	0.051	0.034	0.031	0.033
No. of observations	37,967	43,279	37,967	43,279
Mean of dependent variable	0.302	0.415	0.112	0.160

Notes: *** significant at 1% level; ** significant at 5% level; * significant at 10% level. All specifications include an intercept, year and region fixed effects, degree of urbanisation, education, marital and activity status and household size. Observations are not weighted. Standard errors clustered at the cohort level in parentheses.
Source: Authors' analysis from national health surveys.

Table A2. Age, immigrant arrival cohort and assimilation effects (OLS estimates) in hospital stays and visits to emergency care (foreign-born population from EU15 countries)

	(I)	(II)	(III)	(IV)
	No. of hospitalisations		No. of visits to emergency care	
	Men	Women	Men	Women
Age effects and interactions				
Age	0.033***	0.020***	0.053***	0.028***
	(0.004)	(0.001)	(0.001)	(0.001)
$Age^2/100$	–0.063***	–0.037***	–0.118***	–0.081***
	(0.007)	(0.001)	(0.001)	(0.001)
Age^3 /10000	0.038***	0.023***	0.076***	0.057***
	(0.004)	(0.001)	(0.001)	(0.001)
Age × migrant	–0.068	–0.050**	–0.013	–0.023
	(0.072)	(0.022)	(0.058)	(0.048)
Age/100 × migrant	0.265	0.087*	0.046	0.022
	(0.248)	(0.045)	(0.118)	(0.099)
Age/10,000 × migrant	–0.224	–0.049	–0.044	–0.003
	(0.204)	(0.029)	(0.075)	(0.064)
Immigrant arrival cohort				
Pre–1996	0.540	0.320**	–0.025	0.048
	(0.505)	(0.138)	(0.327)	(0.313)
1996–2007	0.506	0.248**	–0.219	–0.094
	(0.557)	(0.123)	(0.319)	(0.295)
2008–2020	0.586	0.206**	–0.283	–0.014
	(0.714)	(0.101)	(0.270)	(0.256)
Time of residence in Spain				
5–9 years	–1.206	0.064	0.149	0.139
	(1.312)	(0.053)	(0.099)	(0.124)
10–14 years	–1.280	0.014	0.076	0.137
	(1.246)	(0.056)	(0.118)	(0.162)
15 or more years	–1.368	0.015	0.023	0.175
	(1.290)	(0.068)	(0.137)	(0.176)
Adjusted R^2	0.011	0.001	0.014	0.013
No. of observations	37,967	43,279	37,967	43,279
Mean of dependent variable	0.123	0.141	0.397	0.538

Notes: *** significant at 1% level; ** significant at 5% level; * significant at 10% level. All specifications include an intercept, year and region fixed effects, degree of urbanisation, education, marital and activity status and household size. Observations are not weighted. Standard errors clustered at the cohort level in parentheses.
Source: Authors' analysis from national health surveys.

Table A3. Age, immigrant arrival cohort and assimilation effects (OLS estimates) in visits to GP and specialist (foreign-born population from European countries other than the EU15)

	(I)	(II)	(III)	(IV)
	No. of visits to GP		No. of visits to specialist	
	Men	Women	Men	Women
Age effects and interactions				
Age	0.024***	–0.007***	0.015***	0.007***
	(0.000)	(0.000)	(0.000)	(0.000)
$Age^2/100$	–0.037***	0.025***	–0.022***	–0.007***
	(0.000)	(0.001)	(0.000)	(0.001)
Age^3 /10000	0.022***	–0.015***	0.010***	0.001***
	(0.000)	(0.000)	(0.000)	(0.000)
Age × migrant	0.024	0.018	–0.017	0.009
	(0.029)	(0.027)	(0.014)	(0.017)
Age/100 × migrant	–0.074	–0.047	0.031	–0.025
	(0.071)	(0.065)	(0.028)	(0.045)
Age/10,000 × migrant	0.062	0.035	–0.023	0.022
	(0.054)	(0.050)	(0.018)	(0.037)
Immigrant arrival cohort				
Pre–1996	0.061	–0.221	0.188*	0.153
	(0.164)	(0.167)	(0.106)	(0.291)
1996–2007	–0.036	–0.227**	0.019	–0.121
	(0.115)	(0.103)	(0.066)	(0.106)
2008–2020	–0.040	–0.252***	0.041	–0.073
	(0.106)	(0.097)	(0.059)	(0.083)
Time of residence in Spain				
5–9 years	–0.009	0.216***	0.051	0.029
	(0.048)	(0.045)	(0.035)	(0.033)
10–14 years	0.026	0.117**	0.091**	0.138***
	(0.061)	(0.047)	(0.039)	(0.043)
15 or more years	0.137*	0.230***	0.112**	0.067
	(0.070)	(0.077)	(0.045)	(0.043)
Adjusted R^2	0.051	0.034	0.031	0.033
No. of observations	38,065	43,480	38,065	43,480
Mean of dependent variable	0.301	0.413	0.111	0.159

Notes: *** significant at 1% level; ** significant at 5% level; * significant at 10% level. All specifications include an intercept, year and region fixed effects, degree of urbanisation, education, marital and activity status and household size. Observations are not weighted. Standard errors clustered at the cohort level in parentheses.
Source: Authors' analysis from national health surveys.

Table A4. Age, immigrant arrival cohort and assimilation effects (OLS estimates) in hospital stays and visits to emergency care (foreign-born population from European countries other than the EU15)

	(I)	(II)	(III)	(IV)
	No. of hospitalisations		No. of visits to emergency care	
	Men	Women	Men	Women
Age effects and interactions				
Age	0.031***	0.021***	0.054***	0.030***
	(0.000)	(0.001)	(0.000)	(0.002)
$Age^2/100$	–0.058***	–0.039***	–0.119***	–0.084***
	(0.000)	(0.001)	(0.001)	(0.003)
Age^3 /10000	0.036***	0.024***	0.077***	0.058***
	(0.000)	(0.001)	(0.000)	(0.002)
Age × migrant	0.026	0.016	0.055	0.090*
	(0.025)	(0.019)	(0.045)	(0.049)
Age/100 × migrant	–0.069	–0.048	–0.126	–0.224*
	(0.071)	(0.051)	(0.110)	(0.124)
Age/10,000 × migrant	0.054	0.038	0.082	0.163
	(0.063)	(0.040)	(0.083)	(0.098)
Immigrant arrival cohort				
Pre–1996	–0.161	–0.168	0.259	–1.169***
	(0.101)	(0.120)	(0.283)	(0.285)
1996–2007	–0.090**	–0.072*	–0.081	–0.418*
	(0.037)	(0.040)	(0.117)	(0.214)
2008–2020	–0.074*	–0.051	–0.282**	–0.499**
	(0.039)	(0.041)	(0.129)	(0.211)
Time of residence in Spain				
5–9 years	0.030	0.067*	–0.043	0.251**
	(0.020)	(0.037)	(0.080)	(0.118)
10–14 years	0.046*	0.056	0.055	0.378***
	(0.026)	(0.041)	(0.092)	(0.127)
15 or more years	0.065**	0.146	0.030	0.733**
	(0.031)	(0.108)	(0.089)	(0.313)
Adjusted R^2	0.009	0.001	0.014	0.013
No. of observations	38,065	43,480	38,065	43,480
Mean of dependent variable	0.118	0.140	0.400	0.542

Notes: *** significant at 1% level; ** significant at 5% level; * significant at 10% level. All specifications include an intercept, year and region fixed effects, degree of urbanisation, education, marital and activity status and household size. Observations are not weighted. Standard errors clustered at the cohort level in parentheses.
Source: Authors' analysis from national health surveys.

Table A5. Age, immigrant arrival cohort and assimilation effects (OLS estimates) in visits to GP and specialist (foreign-born population from Latin America and the Caribbean)

	(I)	(II)	(III)	(IV)
	No. of visits to GP		No. of visits to specialist	
	Men	Women	Men	Women
Age effects and interactions				
Age	0.023***	–0.005***	0.015***	0.008***
	(0.001)	(0.002)	(0.001)	(0.001)
$Age^2/100$	–0.035***	0.021***	–0.022***	–0.009***
	(0.002)	(0.003)	(0.001)	(0.002)
Age^3 /10000	0.021***	–0.013***	0.010***	0.002**
	(0.001)	(0.002)	(0.001)	(0.001)
Age × migrant	–0.016	0.072***	0.003	0.011
	(0.018)	(0.021)	(0.013)	(0.009)
Age/100 × migrant	0.038	–0.152***	–0.008	–0.025
	(0.044)	(0.049)	(0.032)	(0.022)
Age/10,000 × migrant	–0.025	0.095***	0.007	0.018
	(0.032)	(0.035)	(0.024)	(0.016)
Immigrant arrival cohort				
Pre–1996	–0.015	–0.327***	–0.001	–0.165***
	(0.069)	(0.114)	(0.048)	(0.053)
1996–2007	0.061	–0.281***	0.020	–0.103***
	(0.059)	(0.073)	(0.027)	(0.037)
2008–2020	0.106**	–0.219***	–0.037	–0.087***
	(0.048)	(0.077)	(0.038)	(0.029)
Time of residence in Spain				
5–9 years	–0.040	0.065*	–0.013	0.024
	(0.051)	(0.035)	(0.035)	(0.023)
10–14 years	–0.029	0.141***	–0.053	0.050
	(0.051)	(0.052)	(0.042)	(0.036)
15 or more years	0.006	0.216***	–0.012	0.096**
	(0.057)	(0.058)	(0.040)	(0.041)
Adjusted R^2	0.050	0.033	0.031	0.033
No. of observations	38,772	44,790	38,772	44,790
Mean of dependent variable	0.300	0.416	0.112	0.160

Notes: *** significant at 1% level; ** significant at 5% level; * significant at 10% level. All specifications include an intercept, year and region fixed effects, degree of urbanisation, education, marital and activity status and household size. Observations are not weighted. Standard errors clustered at the cohort level in parentheses.
Source: Authors' analysis from national health surveys.

Table A6. Age, immigrant arrival cohort and assimilation effects (OLS estimates) in hospital stays and visits to emergency care (foreign-born population from Latin America and the Caribbean)

	(I)	(II)	(III)	(IV)
	No. of hospitalisations		No. of visits to emergency care	
	Men	Women	Men	Women
Age effects and interactions				
Age	0.030***	0.021***	0.052***	0.030***
	(0.001)	(0.001)	(0.002)	(0.002)
$Age^2/100$	–0.056***	–0.040***	–0.115***	–0.083***
	(0.002)	(0.003)	(0.004)	(0.003)
Age^3 /10000	0.035***	0.024***	0.075***	0.058***
	(0.001)	(0.001)	(0.002)	(0.002)
Age × migrant	0.008	–0.024*	0.003	0.038
	(0.009)	(0.014)	(0.025)	(0.030)
Age/100 × migrant	–0.017	0.048*	–0.001	–0.067
	(0.023)	(0.027)	(0.056)	(0.058)
Age/10,000 × migrant	0.010	–0.031*	–0.004	0.034
	(0.017)	(0.017)	(0.039)	(0.036)
Immigrant arrival cohort				
Pre–1996	–0.125***	0.204	–0.120	–0.229
	(0.035)	(0.126)	(0.118)	(0.245)
1996–2007	–0.075***	0.147*	–0.018	–0.229
	(0.027)	(0.077)	(0.101)	(0.196)
2008–2020	–0.031	0.145**	0.004	–0.152
	(0.025)	(0.069)	(0.084)	(0.171)
Time of residence in Spain				
5–9 years	0.076***	–0.042	–0.000	0.020
	(0.019)	(0.036)	(0.072)	(0.100)
10–14 years	0.061**	–0.057	0.059	0.116
	(0.024)	(0.035)	(0.075)	(0.114)
15 or more years	0.062**	0.009	–0.004	0.188
	(0.026)	(0.070)	(0.074)	(0.129)
Adjusted R^2	0.009	0.001	0.014	0.013
No. of observations	38,772	44,790	38,772	44,790
Mean of dependent variable	0.118	0.141	0.402	0.549

Notes: *** significant at 1% level; ** significant at 5% level; * significant at 10% level. All specifications include an intercept, year and region fixed effects, degree of urbanisation, education, marital and activity status and household size. Observations are not weighted. Standard errors clustered at the cohort level in parentheses.
Source: Authors' analysis from national health surveys.

Table A7. Age, immigrant arrival cohort and assimilation effects (OLS estimates) in visits to GP and specialist (foreign-born population from Africa)

	(I)	(II)	(III)	(IV)
	No. of visits to GP		No. of visits to specialist	
	Men	Women	Men	Women
Age effects and interactions				
Age	0.024***	–0.007***	0.015***	0.007***
	(0.000)	(0.000)	(0.000)	(0.000)
$Age^2/100$	–0.037***	0.024***	–0.022***	–0.007***
	(0.001)	(0.001)	(0.001)	(0.001)
Age^3 /10000	0.023***	–0.015***	0.010***	0.001***
	(0.000)	(0.001)	(0.000)	(0.000)
Age × migrant	–0.013	–0.057*	–0.005	–0.009
	(0.025)	(0.034)	(0.007)	(0.018)
Age/100 × migrant	0.022	0.111	0.006	0.019
	(0.057)	(0.077)	(0.015)	(0.042)
Age/10,000 × migrant	–0.014	–0.070	–0.004	–0.011
	(0.039)	(0.053)	(0.010)	(0.028)
Immigrant arrival cohort				
Pre–1996	0.017	0.206	–0.046	0.288
	(0.096)	(0.151)	(0.056)	(0.198)
1996–2007	0.088	0.259**	–0.016	0.218
	(0.094)	(0.125)	(0.050)	(0.184)
2008–2020	0.107	0.152	0.016	0.101
	(0.095)	(0.113)	(0.043)	(0.113)
Time of residence in Spain				
5–9 years	–0.017	0.064	–0.029	–0.188
	(0.090)	(0.086)	(0.051)	(0.150)
10–14 years	0.033	0.175	–0.003	–0.176
	(0.102)	(0.120)	(0.041)	(0.172)
15 or more years	0.077	0.188	0.063	–0.282
	(0.110)	(0.125)	(0.052)	(0.189)
Adjusted R^2	0.050	0.033	0.032	0.033
No. of observations	38,245	43,327	38,245	43,327
Mean of dependent variable	0.302	0.418	0.110	0.159

Notes: *** significant at 1% level; ** significant at 5% level; * significant at 10% level. All specifications include an intercept, year and region fixed effects, degree of urbanisation, education, marital and activity status and household size. Observations are not weighted. Standard errors clustered at the cohort level in parentheses.
Source: Authors' analysis from national health surveys.

Table A8. Age, immigrant arrival cohort and assimilation effects (OLS estimates) in hospital stays and visits to emergency care (foreign-born population from Africa)

	(I)	(II)	(III)	(IV)
	No. of hospitalisations		No. of visits to emergency care	
	Men	Women	Men	Women
Age effects and interactions				
Age	0.030***	0.021***	0.053***	0.028***
	(0.000)	(0.002)	(0.001)	(0.001)
$Age^2/100$	–0.057***	–0.040***	–0.118***	–0.079***
	(0.001)	(0.003)	(0.002)	(0.003)
Age^3 /10000	0.035***	0.025***	0.076***	0.056***
	(0.000)	(0.002)	(0.001)	(0.001)
Age × migrant	0.004	–0.051	–0.050	0.090
	(0.029)	(0.046)	(0.053)	(0.065)
Age/100 × migrant	–0.023	0.043	0.097	–0.195
	(0.075)	(0.122)	(0.127)	(0.147)
Age/10,000 × migrant	0.023	0.000	–0.068	0.132
	(0.057)	(0.098)	(0.086)	(0.100)
Immigrant arrival cohort				
Pre–1996	–0.007	1.015	0.238	–0.069
	(0.081)	(0.635)	(0.236)	(0.286)
1996–2007	–0.019	0.786	–0.076	–0.062
	(0.072)	(0.519)	(0.162)	(0.244)
2008–2020	–0.018	0.493	0.097	–0.426**
	(0.058)	(0.353)	(0.190)	(0.204)
Time of residence in Spain				
5–9 years	0.079**	–0.140	0.297**	–0.120
	(0.033)	(0.117)	(0.134)	(0.202)
10–14 years	0.050	0.418	0.386***	–0.119
	(0.034)	(0.492)	(0.097)	(0.167)
15 or more years	0.055	–0.123	0.421***	–0.133
	(0.034)	(0.176)	(0.119)	(0.240)
Adjusted R^2	0.009	0.002	0.014	0.013
No. of observations	38,245	43,327	38,245	43,327
Mean of dependent variable	0.118	0.147	0.404	0.544

Notes: *** significant at 1% level; ** significant at 5% level; * significant at 10% level. All specifications include an intercept, year and region fixed effects, degree of urbanisation, education, marital and activity status and household size. Observations are not weighted. Standard errors clustered at the cohort level in parentheses.
Source: Authors' analysis from national health surveys.

Table A9. Age, immigrant arrival cohort and assimilation effects (OLS estimates) in visits to GP and specialist (low-educated population)

	(I)	(II)	(III)	(IV)
	No. of visits to GP		No. of visits to specialist	
	Men	Women	Men	Women
Age effects and interactions				
Age	0.018***	–0.012***	0.015***	0.003***
	(0.001)	(0.002)	(0.001)	(0.001)
$Age^2/100$	–0.026***	0.038***	–0.024***	–0.001
	(0.003)	(0.004)	(0.002)	(0.001)
Age^3 /10000	0.017***	–0.024***	0.011***	–0.003***
	(0.001)	(0.002)	(0.001)	(0.001)
Age × migrant	–0.007	0.066***	–0.008	0.002
	(0.012)	(0.020)	(0.008)	(0.008)
Age/100 × migrant	0.013	–0.151***	0.015	–0.005
	(0.029)	(0.045)	(0.018)	(0.017)
Age/10,000 × migrant	–0.010	0.098***	–0.010	0.003
	(0.021)	(0.031)	(0.012)	(0.012)
Immigrant arrival cohort				
Pre–1996	–0.141**	–0.258**	–0.015	–0.020
	(0.054)	(0.101)	(0.052)	(0.048)
1996–2007	–0.027	–0.217***	0.022	–0.061*
	(0.041)	(0.074)	(0.047)	(0.036)
2008–2020	–0.018	–0.213***	0.005	–0.066**
	(0.041)	(0.068)	(0.034)	(0.031)
Time of residence in Spain				
5–9 years	0.007	0.052	–0.057	–0.001
	(0.036)	(0.033)	(0.045)	(0.027)
10–14 years	0.072**	0.133***	–0.006	0.056
	(0.032)	(0.040)	(0.040)	(0.035)
15 or more years	0.130***	0.208***	0.025	0.032
	(0.040)	(0.055)	(0.045)	(0.028)
Adjusted R^2	0.050	0.025	0.031	0.031
No. of observations	25,311	29,926	25,311	29,926
Mean of dependent variable	0.329	0.477	0.107	0.150

Notes: *** significant at 1% level; ** significant at 5% level; * significant at 10% level. All specifications include an intercept, year and region fixed effects, degree of urbanisation, education, marital and activity status and household size. Observations are not weighted. Standard errors clustered at the cohort level in parentheses.
Source: Authors' analysis from national health surveys.

Table A10. Age, immigrant arrival cohort and assimilation effects (OLS estimates) in hospital stays and visits to emergency care (low-educated population)

	(I)	(II)	(III)	(IV)
	No. of hospitalisations		No. of visits to emergency care	
	Men	Women	Men	Women
Age effects and interactions				
Age	0.037***	0.027***	0.064***	0.037***
	(0.002)	(0.002)	(0.003)	(0.002)
$Age^2/100$	–0.071***	–0.052***	–0.141***	–0.098***
	(0.003)	(0.003)	(0.006)	(0.004)
Age^3 /10000	0.043***	0.031***	0.090***	0.067***
	(0.002)	(0.002)	(0.003)	(0.002)
Age × migrant	–0.011	–0.031**	–0.039**	0.037
	(0.014)	(0.012)	(0.019)	(0.028)
Age/100 × migrant	0.025	0.064**	0.085*	–0.074
	(0.035)	(0.029)	(0.046)	(0.063)
Age/10,000 × migrant	–0.017	–0.041**	–0.061*	0.046
	(0.026)	(0.020)	(0.033)	(0.043)
Immigrant arrival cohort				
Pre–1996	–0.046	0.232**	0.030	–0.189
	(0.046)	(0.104)	(0.132)	(0.176)
1996–2007	–0.033	0.126**	–0.032	–0.114
	(0.043)	(0.058)	(0.080)	(0.155)
2008–2020	–0.044**	0.108*	–0.082	–0.271***
	(0.021)	(0.058)	(0.074)	(0.102)
Time of residence in Spain				
5–9 years	0.074**	–0.043	0.099	–0.240*
	(0.031)	(0.046)	(0.067)	(0.121)
10–14 years	0.060*	–0.065	0.262***	–0.030
	(0.033)	(0.042)	(0.059)	(0.097)
15 or more years	0.040	–0.020	0.202***	0.013
	(0.035)	(0.064)	(0.066)	(0.135)
Adjusted R^2	0.007	0.002	0.012	0.011
No. of observations	25,311	29,926	25,311	29,926
Mean of dependent variable	0.137	0.145	0.460	0.597

Notes: *** significant at 1% level; ** significant at 5% level; * significant at 10% level. All specifications include an intercept, year and region fixed effects, degree of urbanisation, education, marital and activity status and household size. Observations are not weighted. Standard errors clustered at the cohort level in parentheses.
Source: Authors' analysis from national health surveys.

Table A11. Age, immigrant arrival cohort and assimilation effects (OLS estimates) in visits to GP and specialist (medium- and highly-educated population)

	(I)	(II)	(III)	(IV)
	No. of visits to GP		No. of visits to specialist	
	Men	Women	Men	Women
Age effects and interactions				
Age	0.035***	0.005***	0.020***	0.018***
	(0.003)	(0.002)	(0.001)	(0.002)
$Age^2/100$	–0.054***	–0.003	–0.030***	–0.030***
	(0.005)	(0.003)	(0.002)	(0.003)
Age^3 /10000	0.031***	0.002	0.014***	0.015***
	(0.002)	(0.002)	(0.001)	(0.001)
Age × migrant	0.032*	–0.005	–0.008	–0.002
	(0.018)	(0.019)	(0.015)	(0.010)
Age/100 × migrant	–0.082**	0.018	0.009	0.001
	(0.040)	(0.041)	(0.032)	(0.021)
Age/10,000 × migrant	0.059**	–0.017	–0.004	0.002
	(0.028)	(0.027)	(0.021)	(0.014)
Immigrant arrival cohort				
Pre–1996	0.039	–0.111	0.069	–0.064
	(0.073)	(0.094)	(0.059)	(0.065)
1996–2007	–0.021	–0.063	0.058	–0.034
	(0.068)	(0.075)	(0.054)	(0.055)
2008–2020	–0.021	–0.049	0.025	–0.031
	(0.061)	(0.071)	(0.069)	(0.037)
Time of residence in Spain				
5–9 years	–0.038	0.094**	0.041	–0.029
	(0.044)	(0.043)	(0.029)	(0.035)
10–14 years	–0.056	0.104**	–0.023	0.018
	(0.038)	(0.045)	(0.048)	(0.042)
15 or more years	–0.016	0.169***	0.025	0.043
	(0.045)	(0.056)	(0.041)	(0.057)
Adjusted R^2	0.034	0.016	0.031	0.036
No. of observations	15,625	17,067	15,625	17,067
Mean of dependent variable	0.238	0.310	0.107	0.164

Notes: *** significant at 1% level; ** significant at 5% level; * significant at 10% level. All specifications include an intercept, year and region fixed effects, degree of urbanisation, education, marital and activity status and household size. Observations are not weighted. Standard errors clustered at the cohort level in parentheses.

Source: Authors' analysis from national health surveys.

Table A12. Age, immigrant arrival cohort and assimilation effects (OLS estimates) in hospital stays and visits to emergency care (medium- and highly-educated population)

	(I)	(II)	(III)	(IV)
	No. of hospitalisations		No. of visits to emergency care	
	Men	Women	Men	Women
Age effects and interactions				
Age	0.030	0.016*	0.026***	0.006
	(0.019)	(0.010)	(0.004)	(0.006)
$Age^2/100$	–0.056	–0.032*	–0.064***	–0.032***
	(0.034)	(0.019)	(0.008)	(0.011)
Age^3 /10000	0.035*	0.021**	0.045***	0.025***
	(0.018)	(0.010)	(0.004)	(0.006)
Age × migrant	–0.061	–0.044	0.018	0.014
	(0.077)	(0.027)	(0.039)	(0.039)
Age/100 × migrant	0.173	0.060	–0.038	–0.037
	(0.210)	(0.041)	(0.084)	(0.073)
Age/10,000 × migrant	–0.129	–0.025	0.019	0.023
	(0.156)	(0.024)	(0.056)	(0.044)
Immigrant arrival cohort				
Pre–1996	0.375	0.379	0.150	–0.285
	(0.387)	(0.291)	(0.150)	(0.232)
1996–2007	0.435	0.397	–0.005	–0.109
	(0.454)	(0.256)	(0.152)	(0.216)
2008–2020	0.466	0.342*	–0.043	–0.073
	(0.473)	(0.204)	(0.147)	(0.207)
Time of residence in Spain				
5–9 years	–0.452	0.022	0.055	0.300***
	(0.482)	(0.036)	(0.073)	(0.099)
10–14 years	–0.514	0.166	–0.004	0.263**
	(0.519)	(0.153)	(0.084)	(0.128)
15 or more years	–0.562	0.098*	–0.014	0.445***
	(0.579)	(0.056)	(0.085)	(0.135)
Adjusted R^2	0.010	0.001	0.009	0.017
No. of observations	15,625	17,067	15,625	17,067
Mean of dependent variable	0.088	0.137	0.315	0.475

Notes: *** significant at 1% level; ** significant at 5% level; * significant at 10% level. All specifications include an intercept, year and region fixed effects, degree of urbanisation, education, marital and activity status and household size. Observations are not weighted. Standard errors clustered at the cohort level in parentheses.
Source: Authors' analysis from national health surveys.

Table A13. Age, immigrant arrival cohort and assimilation effects (OLS estimates) in visits to GP and specialist (differences in assimilation in health care utilisation by immigrant arrival cohort)

	(I)	(II)	(III)	(IV)
	No. of visits to GP		No. of visits to specialist	
	Men	Women	Men	Women
Age effects and interactions				
Age	0.023***	–0.006***	0.014***	0.007***
	(0.002)	(0.001)	(0.001)	(0.001)
$Age^2/100$	–0.034***	0.023***	–0.021***	–0.008***
	(0.003)	(0.002)	(0.002)	(0.001)
Age^3 /10000	0.021***	–0.014***	0.009***	0.002***
	(0.002)	(0.001)	(0.001)	(0.001)
Age × migrant	0.006	0.041***	–0.005	–0.002
	(0.010)	(0.015)	(0.006)	(0.006)
Age/100 × migrant	–0.020	–0.088***	0.006	0.003
	(0.023)	(0.033)	(0.014)	(0.013)
Age/10,000 × migrant	0.014	0.052**	–0.004	–0.003
	(0.017)	(0.023)	(0.010)	(0.009)
Immigrant arrival cohort				
Pre–1996	0.079	–0.157*	0.009	–0.096**
	(0.067)	(0.091)	(0.050)	(0.045)
1996–2007	–0.085**	–0.225***	–0.048	–0.095**
	(0.039)	(0.079)	(0.031)	(0.041)
2008–2020	0.016	–0.168***	–0.015	–0.059**
	(0.032)	(0.052)	(0.026)	(0.024)
Time of residence in Spain				
Years × Pre-1996	–0.001	0.004	0.001	0.003**
	(0.002)	(0.002)	(0.001)	(0.001)
Years × 1996–2007	0.009***	0.013***	0.006***	0.006**
	(0.002)	(0.004)	(0.002)	(0.003)
Years × 2008–2020	–0.002	0.009*	0.002	0.003
	(0.005)	(0.005)	(0.002)	(0.002)
Adjusted R^2	0.050	0.033	0.031	0.033
No. of observations	40,936	46,993	40,936	46,993
Mean of dependent variable	0.293	0.410	0.107	0.156

Notes: *** significant at 1% level; ** significant at 5% level; * significant at 10% level. All specifications include an intercept, year and region fixed effects, degree of urbanisation, education, marital and activity status and household size. Observations are not weighted. Standard errors clustered at the cohort level in parentheses.
Source: Authors' analysis from national health surveys.

Table A14. Age, immigrant arrival cohort and assimilation effects (OLS estimates) in hospital stays and visits to emergency care (differences in assimilation in health care utilisation by immigrant arrival cohort)

	(I)	(II)	(III)	(IV)
	No. of hospitalisations		No. of visits to emergency care	
	Men	Women	Men	Women
Age effects and interactions				
Age	0.033***	0.023***	0.050***	0.029***
	(0.005)	(0.003)	(0.003)	(0.002)
$Age^2/100$	–0.063***	–0.044***	–0.113***	–0.081***
	(0.009)	(0.006)	(0.007)	(0.004)
Age^3 /10000	0.038***	0.027***	0.073***	0.057***
	(0.005)	(0.004)	(0.004)	(0.002)
Age × migrant	–0.038	–0.019*	–0.016	0.044**
	(0.038)	(0.011)	(0.018)	(0.021)
Age/100 × migrant	0.100	0.027	0.033	–0.092**
	(0.103)	(0.029)	(0.040)	(0.044)
Age/10,000 × migrant	–0.072	–0.013	–0.028	0.056*
	(0.074)	(0.021)	(0.027)	(0.030)
Immigrant arrival cohort				
Pre–1996	–0.089	0.246***	0.120	–0.137
	(0.070)	(0.092)	(0.151)	(0.146)
1996–2007	0.084	0.226*	–0.003	–0.290**
	(0.070)	(0.135)	(0.077)	(0.119)
2008–2020	0.276	0.142***	–0.043	–0.295***
	(0.308)	(0.046)	(0.082)	(0.106)
Time of residence in Spain				
Years × Pre-1996	0.000	–0.000	0.003	0.001
	(0.002)	(0.002)	(0.003)	(0.003)
Years × 1996–2007	–0.007	–0.002	0.010**	0.017**
	(0.006)	(0.006)	(0.004)	(0.007)
Years × 2008–2020	–0.024	–0.001	0.014**	0.014
	(0.031)	(0.004)	(0.007)	(0.010)
Adjusted R^2	0.008	0.001	0.013	0.013
No. of observations	40,936	46,993	40,936	46,993
Mean of dependent variable	0.118	0.142	0.403	0.548

Notes: *** significant at 1% level; ** significant at 5% level; * significant at 10% level. All specifications include an intercept, year and region fixed effects, degree of urbanisation, education, marital and activity status and household size. Observations are not weighted. Standard errors clustered at the cohort level in parentheses.
Source: Authors' analysis from national health surveys.

Figure A1. Differences in health care use between 35-year-old migrants after 15 years in Spain by arrival cohort (foreign-born population from EU15 countries)

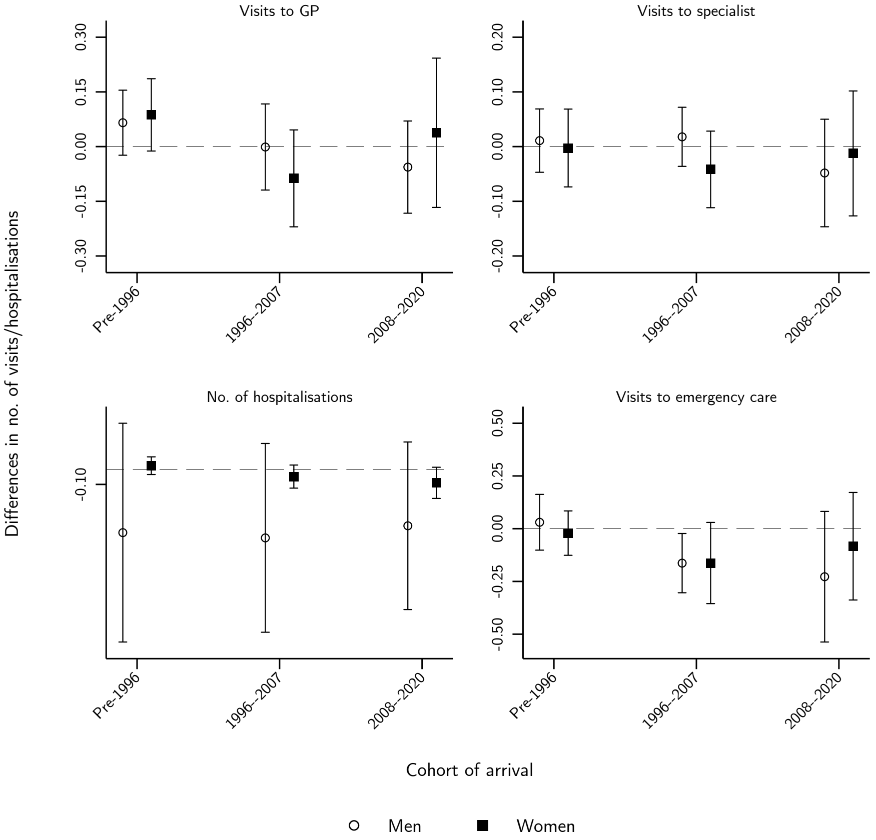

Note: The graph shows point estimates and 90%-level confidence intervals. We assume that migrants enter the country 15 years ago or earlier.
Source: Authors' analysis from results in Table A1 and A2.

Figure A2. Differences in health care use between between 35-year-old migrants after 15 years in Spain by arrival cohort (foreign-born population from European countries other than EU15)

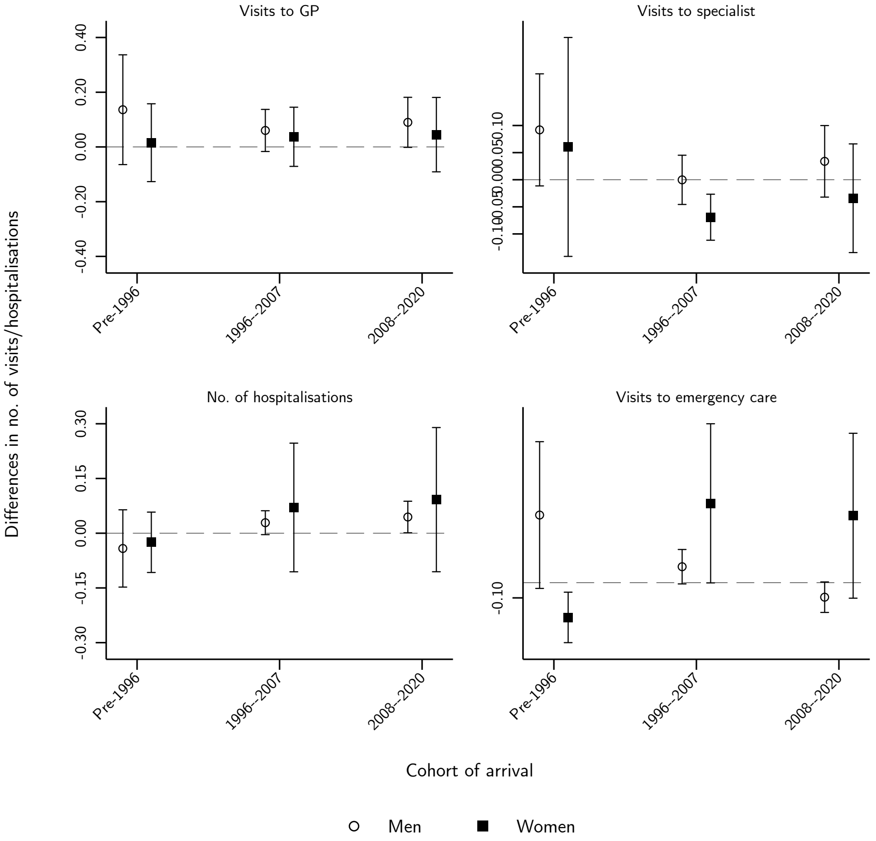

Note: The graph shows point estimates and 90%-level confidence intervals. We assume that migrants enter the country 15 years ago or earlier.
Source: Authors' analysis from results in Table A3 and A4.

Figure A3. Differences in health care use between 35-year-old migrants after 15 years in Spainby arrival cohort (foreign-born population from Latin America and the Caribbean)

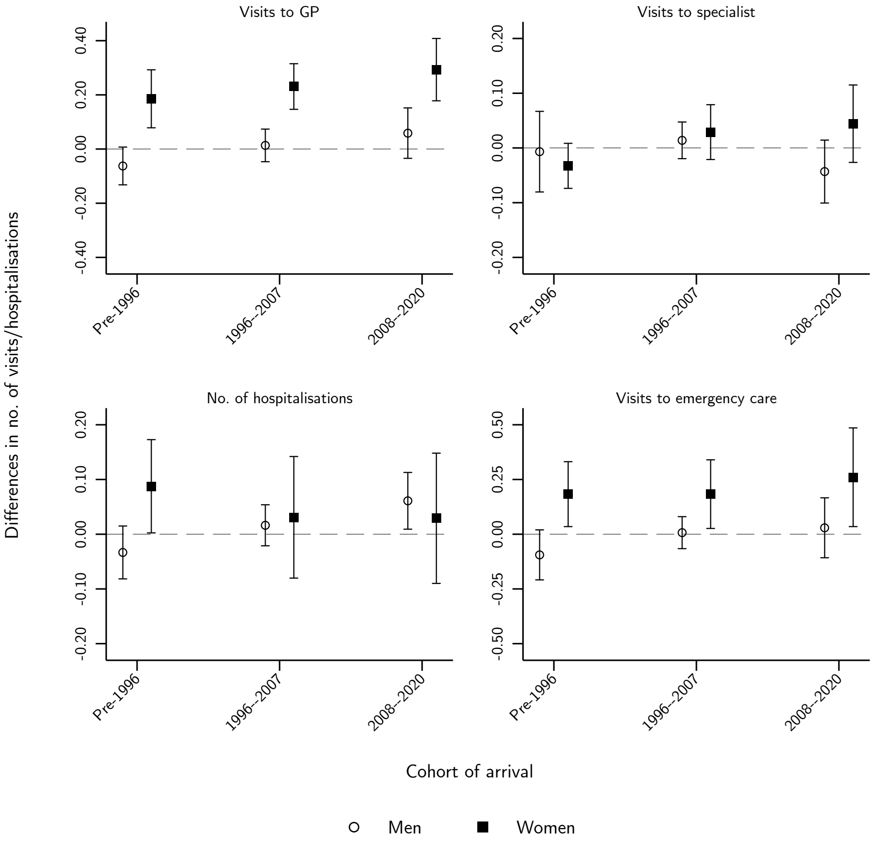

Note: The graph shows point estimates and 90%-level confidence intervals. We assume that migrants enter the country 15 years ago or earlier.
Source: Authors' analysis from results in Table A5 and A6.

Figure A4. Differences in health care use between 35-year-old migrants after 15 years in Spain by arrival cohort (foreign-born population from Africa)

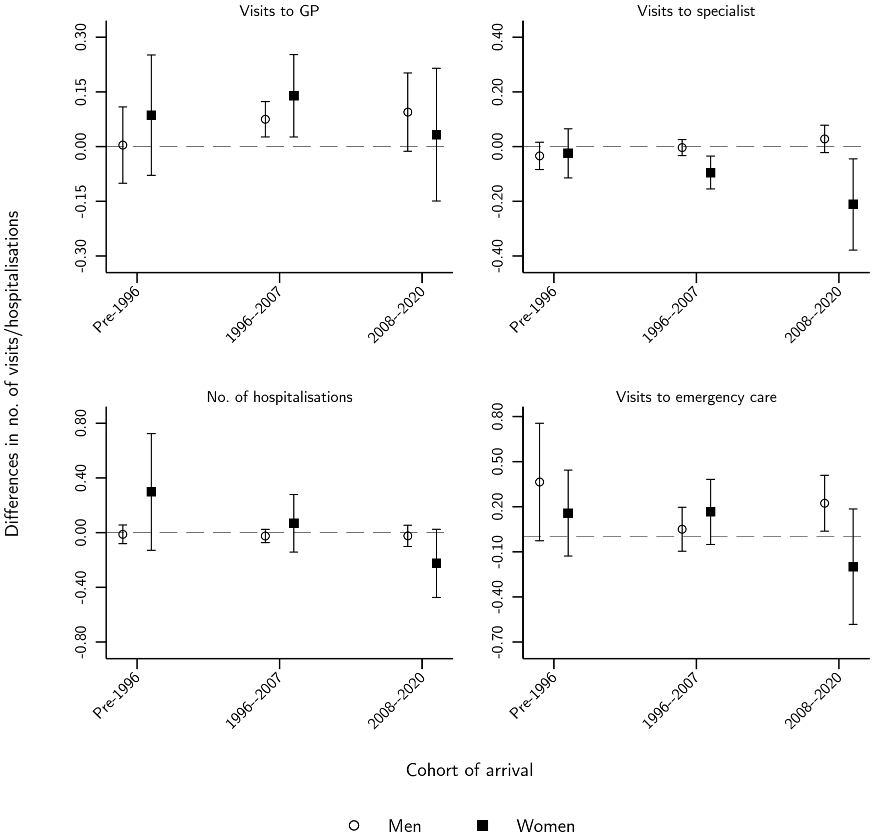

Note: The graph shows point estimates and 90%-level confidence intervals. We assume that migrants enter the country 15 years ago or earlier.
Source: Authors' analysis from results in Table A7 and A8.

Figure A5. Differences in health care use between 35-year-old migrants after 15 years in Spainby arrival cohort (foreign-born low-educated population)

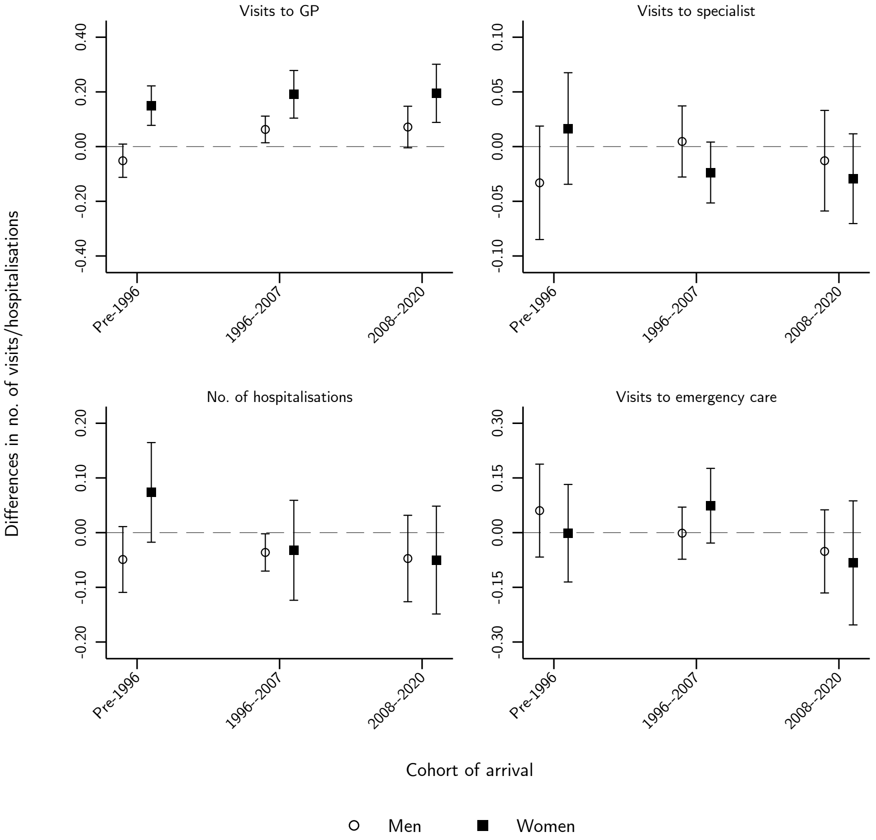

Note: The graph shows point estimates and 90%-level confidence intervals. We assume that migrants enter the country 15 years ago or earlier.
Source: Authors' analysis from results in Table A9 and A10.

Figure A6. Differences in health care use between 35-year-old migrants after 15 years in Spain by arrival cohort (foreign-born medium and highly-educated population)

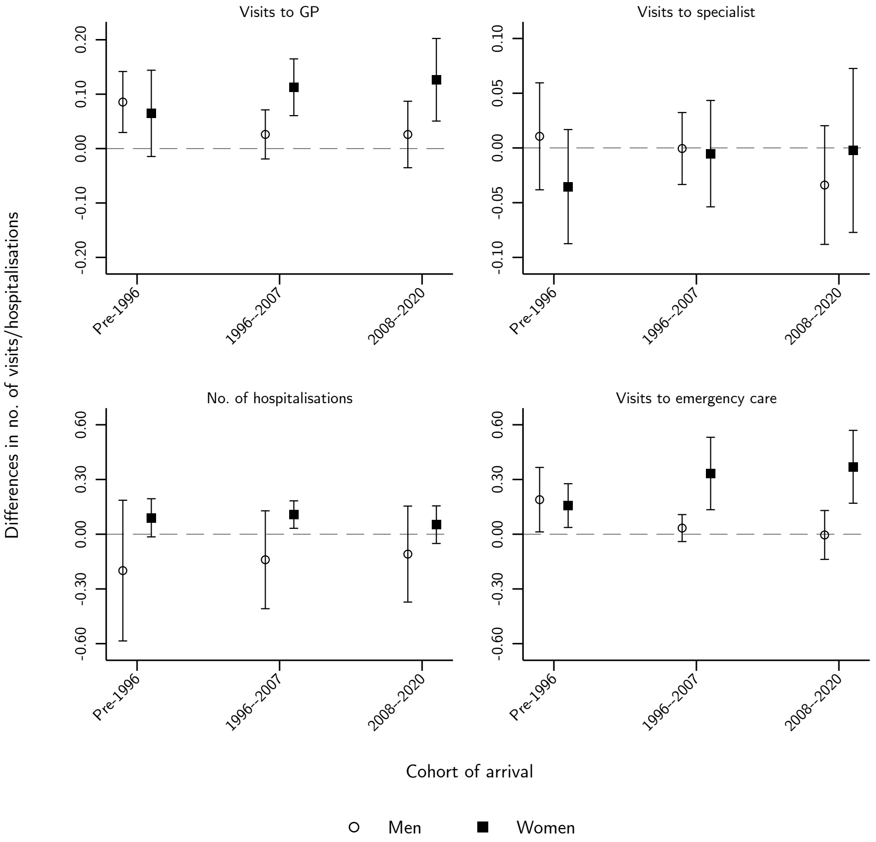

Note: The graph shows point estimates and 90%-level confidence intervals. We assume that migrants enter the country 15 years ago or earlier.
Source: Authors' analysis from results in Table A11 and A12.

Table A15. Age, immigrant arrival cohort and assimilation effects (OLS estimates) in obesity and alcohol consumption

	(I)	(II)	(III)	(IV)
	Obesity		Alcohol consumption	
	Men	Women	Men	Women
Immigrant arrival cohort				
Pre–1996	0.029	–0.061**	0.004	0.119***
	(0.026)	(0.029)	(0.055)	(0.045)
1996–2007	0.024	–0.048**	–0.041	0.049
	(0.020)	(0.023)	(0.043)	(0.039)
2008–2020	0.009	–0.052*	–0.020	0.054*
	(0.019)	(0.026)	(0.040)	(0.032)
Time of residence in Spain				
5–9 years	–0.012	0.018	–0.059	–0.059**
	(0.015)	(0.018)	(0.037)	(0.027)
10–14 years	–0.018	0.009	–0.070*	–0.045
	(0.018)	(0.020)	(0.038)	(0.034)
15 or more years	0.015	–0.006	–0.047	–0.042
	(0.019)	(0.023)	(0.044)	(0.034)
Adjusted R^2	0.036	0.051	0.074	0.066
No. of observations	39,508	43,348	31,308	35,679
Mean of dependent variable	0.169	0.158	0.486	0.246

Notes: *** significant at 1% level; ** significant at 5% level; * significant at 10% level. Obesity is defined as BMI of 30 or higher. Alcohol consumption is a binary variable that takes the value one if the person drank at least 1–2 times alcohol in the last week and zero otherwise. All specifications include an intercept, year and region fixed effects and controls for age (introduced though a third-degree polynomial fully interacted with migrant status), degree of urbanisation, education, and marital and activity status and household size. Observations are not weighted. Standard errors clustered at the cohort level in parentheses.
Source: Authors' analysis from national health surveys.

Table A16. Age, immigrant arrival cohort and assimilation effects (OLS estimates) in smoking behaviour and physical exercise

	(I)	(II)	(III)	(IV)
	Smoking behaviour		Physical exercise	
	Men	Women	Men	Women
Pre–1996	0.045	–0.011	0.023	0.172***
	(0.049)	(0.030)	(0.040)	(0.047)
1996–2007	0.006	–0.056**	0.003	0.052
	(0.042)	(0.027)	(0.034)	(0.041)
2008–2020	–0.001	–0.057**	–0.016	–0.010
	(0.033)	(0.026)	(0.031)	(0.030)
Time of residence in Spain				
5–9 years	–0.000	0.010	–0.020	–0.020
	(0.026)	(0.026)	(0.032)	(0.034)
10–14 years	0.005	–0.004	–0.019	–0.029
	(0.029)	(0.024)	(0.029)	(0.033)
15 or more years	0.038	–0.009	–0.031	–0.061
	(0.026)	(0.025)	(0.039)	(0.042)
Adjusted R^2	0.087	0.053	0.083	0.107
No. of observations	40,897	46,953	40,874	46,951
Mean of dependent variable	0.160	0.101	0.290	0.207

Notes: *** significant at 1% level; ** significant at 5% level; * significant at 10% level. Smoking behaviour is a binary variable that takes the value one if the person smokes and zero otherwise. Physical exercise is a binary variable whose value is one if the interviewed person does physical exercise several times a week and zero otherwise. All specifications include an intercept, year and region fixed effects, degree of urbanisation, education, marital and activity status and household size. Observations are not weighted. Standard errors clustered at the cohort level in parentheses.
Source: Authors' analysis from national health surveys.

Table A17. Robustness checks (I): Immigrant arrival cohort and assimilation effects (OLS estimates) in visits to GP and specialist (alternative non-linear specification)

	(I)	(II)	(III)	(IV)
	No. of visits to GP		No. of visits to specialist	
	Men	Women	Men	Women
Age effects and interactions				
Age	0.023***	–0.006***	0.014***	0.007***
	(0.002)	(0.001)	(0.001)	(0.001)
$Age^2/100$	–0.034***	0.023***	–0.021***	–0.008***
	(0.003)	(0.002)	(0.002)	(0.001)
Age^3 /10000	0.021***	–0.014***	0.009***	0.002***
	(0.002)	(0.001)	(0.001)	(0.001)
Age × migrant	0.006	0.044***	–0.004	0.000
	(0.010)	(0.015)	(0.007)	(0.006)
Age/100 × migrant	–0.017	–0.095***	0.005	–0.002
	(0.024)	(0.033)	(0.015)	(0.013)
Age/10,000 × migrant	0.012	0.058**	–0.003	0.002
	(0.017)	(0.023)	(0.010)	(0.009)
Immigrant arrival cohort				
Pre–1996	–0.050	–0.182*	–0.029	–0.076*
	(0.059)	(0.093)	(0.028)	(0.043)
1996–2007	–0.025	–0.150**	–0.014	–0.068*
	(0.054)	(0.074)	(0.025)	(0.035)
2008–2020	–0.014	–0.148**	–0.016	–0.063**
	(0.037)	(0.062)	(0.026)	(0.030)
Time of residence in Spain				
3–7 years	0.033	0.011	0.011	0.015
	(0.039)	(0.052)	(0.023)	(0.029)
8–12 years	–0.030	0.025	0.001	0.006
	(0.023)	(0.027)	(0.018)	(0.023)
13 or more years	0.040	0.121***	0.053***	0.057**
	(0.031)	(0.035)	(0.019)	(0.028)
Adjusted R^2	0.050	0.033	0.031	0.033
No. of observations	40,936	46,993	40,936	46,993
Mean of dependent variable	0.293	0.410	0.107	0.156

Notes: *** significant at 1% level; ** significant at 5% level; * significant at 10% level. All specifications include an intercept, year and region fixed effects, degree of urbanisation, education, marital and activity status and household size. Observations are not weighted. Standard errors clustered at the cohort level in parentheses.
Source: Authors' analysis from national health surveys.

Table A18. Robustness checks (I): Immigrant arrival cohort and assimilation effects (OLS estimates) in hospital stays and visits to emergency care (alternative nonlinear specification)

	(I)	(II)	(III)	(IV)
	No. of hospitalisations		No. of visits to emergency care	
	Men	Women	Men	Women
Age effects and interactions				
Age	0.033***	0.023***	0.050***	0.029***
	(0.005)	(0.003)	(0.003)	(0.002)
$Age^2/100$	–0.063***	–0.044***	–0.113***	–0.081***
	(0.009)	(0.006)	(0.007)	(0.004)
Age^3 /10000	0.038***	0.027***	0.073***	0.057***
	(0.005)	(0.004)	(0.004)	(0.002)
Age × migrant	–0.040	–0.020*	–0.016	0.046**
	(0.041)	(0.011)	(0.018)	(0.020)
Age/100 × migrant	0.103	0.029	0.033	–0.094**
	(0.108)	(0.029)	(0.040)	(0.043)
Age/10,000 × migrant	–0.074	–0.014	–0.027	0.057**
	(0.078)	(0.022)	(0.028)	(0.028)
Immigrant arrival cohort				
Pre–1996	–0.196	0.269**	–0.029	–0.322**
	(0.182)	(0.118)	(0.096)	(0.141)
1996–2007	–0.145	0.195**	–0.104	–0.230*
	(0.140)	(0.092)	(0.085)	(0.118)
2008–2020	–0.002	0.124**	–0.090	–0.276***
	(0.039)	(0.054)	(0.088)	(0.102)
Time of residence in Spain				
3–7 years	0.264	0.001	0.130**	0.034
	(0.235)	(0.040)	(0.064)	(0.078)
8–12 years	–0.112	0.045	0.095***	0.070
	(0.131)	(0.075)	(0.033)	(0.055)
13 or more years	–0.120	–0.025	0.127***	0.178**
	(0.133)	(0.037)	(0.039)	(0.085)
Adjusted R^2	0.008	0.001	0.014	0.013
No. of observations	40,936	46,993	40,936	46,993
Mean of dependent variable	0.118	0.142	0.403	0.548

Notes: *** significant at 1% level; ** significant at 5% level; * significant at 10% level. All specifications include an intercept, year and region fixed effects, degree of urbanisation, education, marital and activity status and household size. Observations are not weighted. Standard errors clustered at the cohort level in parentheses.
Source: Authors' analysis from national health surveys.

Table A19. Robustness checks (I): Immigrant arrival cohort and assimilation effects (OLS estimates) in visits to GP and specialist (linear specification)

	(I)	(II)	(III)	(IV)
	No. of visits to GP		No. of visits to specialist	
	Men	Women	Men	Women
Age effects and interactions				
Age	0.023***	–0.006***	0.014***	0.007***
	(0.002)	(0.001)	(0.001)	(0.001)
$Age^2/100$	–0.034***	0.023***	–0.021***	–0.008***
	(0.003)	(0.002)	(0.002)	(0.001)
Age^3 /10000	0.021***	–0.014***	0.009***	0.002***
	(0.002)	(0.001)	(0.001)	(0.001)
Age × migrant	0.004	0.040***	–0.006	–0.002
	(0.010)	(0.014)	(0.007)	(0.006)
Age/100 × migrant	–0.012	–0.081**	0.010	0.006
	(0.023)	(0.032)	(0.015)	(0.014)
Age/10,000 × migrant	0.008	0.046**	–0.007	–0.005
	(0.016)	(0.022)	(0.010)	(0.010)
Immigrant arrival cohort				
Pre–1996	–0.019	–0.258***	–0.042	–0.126***
	(0.055)	(0.094)	(0.038)	(0.043)
1996–2007	0.002	–0.143**	–0.005	–0.064**
	(0.033)	(0.059)	(0.021)	(0.025)
2008–2020	–0.005	–0.161***	–0.020	–0.066***
	(0.026)	(0.054)	(0.022)	(0.020)
Time of residence in Spain				
Years since migration	0.001	0.006***	0.002**	0.004***
	(0.002)	(0.002)	(0.001)	(0.001)
Adjusted R^2	0.050	0.033	0.031	0.033
No. of observations	40,936	46,993	40,936	46,993
Mean of dependent variable	0.293	0.410	0.107	0.156

Notes: *** significant at 1% level; ** significant at 5% level; * significant at 10% level. All specifications include an intercept, year and region fixed effects, degree of urbanisation, education, marital and activity status and household size. Observations are not weighted. Standard errors clustered at the cohort level in parentheses.

Source: Authors' analysis from national health surveys.

Table A20. Robustness checks (I): Immigrant arrival cohort and assimilation effects (OLS estimates) in hospital stays and visits to emergency care (linear specification)

	(I)	(II)	(III)	(IV)
	No. of hospitalisations		No. of visits to emergency care	
	Men	Women	Men	Women
Age effects and interactions				
Age	0.033***	0.023***	0.050***	0.029***
	(0.005)	(0.003)	(0.003)	(0.002)
$Age^2/100$	–0.063***	–0.044***	–0.113***	–0.081***
	(0.009)	(0.006)	(0.007)	(0.004)
Age^3 /10000	0.038***	0.027***	0.073***	0.057***
	(0.005)	(0.004)	(0.004)	(0.002)
Age × migrant	–0.038	–0.019	–0.016	0.042**
	(0.039)	(0.012)	(0.018)	(0.020)
Age/100 × migrant	0.097	0.026	0.037	–0.081*
	(0.103)	(0.031)	(0.040)	(0.043)
Age/10,000 × migrant	–0.069	–0.011	–0.032	0.046
	(0.073)	(0.023)	(0.027)	(0.029)
Immigrant arrival cohort				
Pre–1996	0.041	0.268**	0.027	–0.315**
	(0.089)	(0.112)	(0.119)	(0.151)
1996–2007	0.052	0.208**	0.042	–0.159
	(0.062)	(0.092)	(0.077)	(0.100)
2008–2020	0.174	0.140***	–0.005	–0.265***
	(0.179)	(0.050)	(0.073)	(0.098)
Time of residence in Spain				
Years since migration	–0.003	–0.001	0.006**	0.006*
	(0.004)	(0.002)	(0.003)	(0.003)
Adjusted R^2	0.007	0.001	0.013	0.013
No. of observations	40,936	46,993	40,936	46,993
Mean of dependent variable	0.118	0.142	0.403	0.548

Notes: *** significant at 1% level; ** significant at 5% level; * significant at 10% level. All specifications include an intercept, year and region fixed effects, degree of urbanisation, education, marital and activity status and household size. Observations are not weighted. Standard errors clustered at the cohort level in parentheses.

Source: Authors' analysis from national health surveys.

Table A21. Robustness checks (II): Immigrant arrival cohort and assimilation effects (OLS estimates) in visits to GP and specialist (including regional linear time trends)

	(I)	(II)	(III)	(IV)
	No. of visits to GP		No. of visits to specialist	
	Men	Women	Men	Women
Age effects and interactions				
Age	0.023***	–0.006***	0.014***	0.007***
	(0.002)	(0.001)	(0.001)	(0.001)
$Age^2/100$	–0.034***	0.023***	–0.021***	–0.008***
	(0.003)	(0.002)	(0.002)	(0.001)
Age^3 /10000	0.021***	–0.014***	0.009***	0.002***
	(0.002)	(0.001)	(0.001)	(0.001)
Age × migrant	0.005	0.043***	–0.005	0.000
	(0.010)	(0.015)	(0.007)	(0.006)
Age/100 × migrant	–0.017	–0.093***	0.007	–0.002
	(0.023)	(0.033)	(0.015)	(0.014)
Age/10,000 × migrant	0.012	0.057**	–0.004	0.002
	(0.016)	(0.023)	(0.010)	(0.009)
Immigrant arrival cohort				
Pre–1996	–0.037	–0.239***	0.000	–0.049
	(0.043)	(0.079)	(0.032)	(0.039)
1996–2007	–0.002	–0.193***	0.018	–0.051*
	(0.038)	(0.055)	(0.029)	(0.028)
2008–2020	0.012	–0.168***	–0.002	–0.049**
	(0.029)	(0.051)	(0.028)	(0.019)
Time of residence in Spain				
5–9 years	–0.017	0.066***	–0.009	–0.014
	(0.027)	(0.023)	(0.029)	(0.022)
10–14 years	0.014	0.116***	–0.010	0.039
	(0.030)	(0.029)	(0.027)	(0.031)
15 or more years	0.068**	0.197***	0.032	0.043
	(0.028)	(0.039)	(0.029)	(0.034)
Adjusted R^2	0.050	0.034	0.031	0.034
No. of observations	40,936	46,993	40,936	46,993
Mean of dependent variable	0.293	0.410	0.107	0.156

Notes: *** significant at 1% level; ** significant at 5% level; * significant at 10% level. All specifications include an intercept, year and region fixed effects, degree of urbanisation, education, marital and activity status, household size and regional linear time trends. Observations are not weighted. Standard errors clustered at the cohort level in parentheses.
Source: Authors' analysis from national health surveys.

Table A22. Robustness checks (II): Immigrant arrival cohort and assimilation effects (OLS estimates) in hospital stays and visits to emergency care (including regional linear time trends)

	(I)	(II)	(III)	(IV)
	No. of hospitalisations		No. of visits to emergency care	
	Men	Women	Men	Women
Age effects and interactions				
Age	0.033***	0.023***	0.051***	0.029***
	(0.005)	(0.003)	(0.003)	(0.002)
$Age^2/100$	–0.063***	–0.043***	–0.113***	–0.082***
	(0.009)	(0.006)	(0.007)	(0.004)
Age^3 /10000	0.038***	0.026***	0.074***	0.058***
	(0.005)	(0.004)	(0.004)	(0.002)
Age × migrant	–0.037	–0.019	–0.018	0.045**
	(0.037)	(0.011)	(0.018)	(0.021)
Age/100 × migrant	0.097	0.026	0.039	–0.094**
	(0.100)	(0.030)	(0.041)	(0.044)
Age/10,000 × migrant	–0.071	–0.012	–0.031	0.058**
	(0.073)	(0.022)	(0.028)	(0.029)
Immigrant arrival cohort				
Pre–1996	0.190	0.231**	0.113	–0.333**
	(0.220)	(0.101)	(0.089)	(0.140)
1996–2007	0.230	0.187***	0.008	–0.213*
	(0.256)	(0.067)	(0.068)	(0.118)
2008–2020	0.262	0.145***	–0.022	–0.255**
	(0.284)	(0.046)	(0.072)	(0.098)
Time of residence in Spain				
5–9 years	–0.209	–0.023	0.074*	0.031
	(0.267)	(0.024)	(0.043)	(0.083)
10–14 years	–0.237	0.036	0.137***	0.130
	(0.277)	(0.071)	(0.050)	(0.096)
15 or more years	–0.272	0.020	0.100**	0.237**
	(0.303)	(0.049)	(0.044)	(0.107)
Adjusted R^2	0.008	0.001	0.014	0.014
No. of observations	40,936	46,993	40,936	46,993
Mean of dependent variable	0.118	0.142	0.403	0.548

Notes: *** significant at 1% level; ** significant at 5% level; * significant at 10% level. All specifications include an intercept, year and region fixed effects, degree of urbanisation, education, marital and activity status, household size and regional linear time trends. Observations are not weighted. Standard errors clustered at the cohort level in parentheses.
Source: Authors' analysis from national health surveys.

Table A23. Robustness checks (I): Immigrant arrival cohort and assimilation effects (Poisson-regression estimates) in visits to GP and specialist

	(III)	(II)	(III)	(IV)
	No. of visits to GP		No. of visits to specialist	
	Men	Women	Men	Women
Age effects and interactions				
Age	0.080***	–0.008***	0.152***	0.056***
	(0.004)	(0.002)	(0.005)	(0.004)
$Age^2/100$	–0.104***	0.048***	–0.239***	–0.068***
	(0.007)	(0.004)	(0.011)	(0.008)
Age^3 /10000	0.052***	–0.033***	0.115***	0.021***
	(0.004)	(0.002)	(0.006)	(0.004)
Age × migrant	–0.001	0.111***	–0.072	0.024
	(0.037)	(0.041)	(0.093)	(0.053)
Age/100 × migrant	–0.020	–0.237***	0.127	–0.050
	(0.080)	(0.086)	(0.205)	(0.115)
Age/10,000 × migrant	0.021	0.146***	–0.076	0.034
	(0.053)	(0.056)	(0.136)	(0.075)
Immigrant arrival cohort				
Pre–1996	0.013	–0.640***	0.545	–0.144
	(0.200)	(0.233)	(0.381)	(0.325)
1996–2007	0.125	–0.536***	0.613*	–0.185
	(0.188)	(0.187)	(0.341)	(0.215)
2008–2020	0.056	–0.525***	–0.423	–0.519**
	(0.133)	(0.179)	(0.426)	(0.204)
Time of residence in Spain				
5–9 years	–0.096	0.228***	–0.378	–0.321*
	(0.152)	(0.072)	(0.284)	(0.182)
10–14 years	0.002	0.338***	–0.656**	–0.000
	(0.153)	(0.088)	(0.271)	(0.199)
15 or more years	0.168	0.516***	–0.220	–0.043
	(0.145)	(0.103)	(0.240)	(0.253)
R^2	0.054	0.035	0.039	0.036
No. of observations	40,936	46,993	40,936	46,993
Mean of dependent variable	0.293	0.410	0.107	0.156

Notes: *** significant at 1% level; ** significant at 5% level; * significant at 10% level. All specifications include an intercept, year and region fixed effects, degree of urbanisation, education, marital and activity status, and household size. Observations are not weighted. Standard errors clustered at the cohort level in parentheses. R^2 is the squared coefficient of correlation between the actual and the fitted values, as suggested by Zheng and Agresti (2000).
Source: Authors' analysis from national health surveys.

Table A24. Robustness checks (III): Immigrant arrival cohort and assimilation effects (Poisson-regression estimates) in hospital stays and visits to emergency care

	(I)	(II)	(III)	(IV)
	No. of hospitalisations		No. of visits to emergency care	
	Men	Women	Men	Women
Age effects and interactions				
Pre–1996	0.798	0.230**	0.163	–0.541**
	(0.713)	(0.101)	(0.200)	(0.240)
1996–2007	0.968	0.185***	–0.099	–0.354*
	(0.717)	(0.067)	(0.151)	(0.203)
2008–2020	1.119*	0.144***	–0.175	–0.420***
	(0.660)	(0.046)	(0.173)	(0.157)
Immigrant arrival cohort				
Pre–1996	0.798	0.230**	0.163	–0.541**
	(0.713)	(0.101)	(0.200)	(0.240)
1996–2007	0.968	0.185***	–0.099	–0.354*
	(0.717)	(0.067)	(0.151)	(0.203)
2008–2020	1.119*	0.144***	–0.175	–0.420***
	(0.660)	(0.046)	(0.173)	(0.157)
Time of residence in Spain				
5–9 years	–1.070	–0.021	0.220*	0.048
	(0.784)	(0.024)	(0.128)	(0.155)
10–14 years	–1.319	0.040	0.369***	0.185
	(0.819)	(0.073)	(0.139)	(0.178)
15 or more years	–1.575*	0.022	0.279**	0.361**
	(0.899)	(0.049)	(0.130)	(0.184)
R^2	0.016	0.002	0.016	0.015
No. of observations	40,936	46,993	40,936	46,993
Mean of dependent variable	0.118	0.142	0.403	0.548

Notes: *** significant at 1% level; ** significant at 5% level; * significant at 10% level. All specifications include an intercept, year and region fixed effects, degree of urbanisation, education, marital and activity status and household size. Observations are not weighted. Standard errors clustered at the cohort level in parentheses. R^2 is the squared coefficient of correlation between the actual and the fitted values, as suggested by Zheng and Agresti (2000).
Source: Authors' analysis from national health surveys.

Table A25. Robustness checks (IV): Immigrant arrival cohort and assimilation effects (OLS estimates) in visits to GP and specialist (population with just NHS coverage)

	(I)	(II)	(III)	(IV)
	No. of visits to GP		No. of visits to specialist	
	Men	Women	Men	Women
Age effects and interactions				
Age	0.023***	–0.005***	0.015***	0.007***
	(0.002)	(0.001)	(0.001)	(0.001)
$Age^2/100$	–0.035***	0.021***	–0.023***	–0.007***
	(0.003)	(0.002)	(0.002)	(0.001)
Age^3 /10000	0.021***	–0.013***	0.010***	0.001*
	(0.002)	(0.001)	(0.001)	(0.001)
Age × migrant	0.002	0.039**	–0.007	0.003
	(0.011)	(0.016)	(0.007)	(0.006)
Age/100 × migrant	–0.006	–0.085**	0.011	–0.009
	(0.027)	(0.036)	(0.016)	(0.013)
Age/10,000 × migrant	0.004	0.052**	–0.007	0.006
	(0.019)	(0.025)	(0.010)	(0.009)
Immigrant arrival cohort				
Pre–1996	–0.035	–0.189**	0.019	–0.030
	(0.050)	(0.088)	(0.030)	(0.039)
1996–2007	0.008	–0.141**	0.034	–0.045
	(0.044)	(0.063)	(0.028)	(0.030)
2008–2020	0.036	–0.140**	0.009	–0.052***
	(0.034)	(0.057)	(0.028)	(0.019)
Time of residence in Spain				
5–9 years	–0.025	0.046*	–0.009	–0.020
	(0.032)	(0.025)	(0.028)	(0.021)
10–14 years	0.004	0.082***	–0.008	0.022
	(0.034)	(0.030)	(0.026)	(0.032)
15 or more years	0.053	0.164***	0.020	0.026
	(0.032)	(0.042)	(0.027)	(0.030)
Adjusted R^2	0.052	0.032	0.032	0.033
No. of observations	36,497	43,294	36,497	43,294
Mean of dependent variable	0.298	0.419	0.104	0.151

Notes: *** significant at 1% level; ** significant at 5% level; * significant at 10% level. All specifications include an intercept, year and region fixed effects, degree of urbanisation, education, marital and activity status and household size. Observations are not weighted. Standard errors clustered at the cohort level in parentheses.
Source: Authors' analysis from national health surveys.

Table A26. Robustness checks (IV): Immigrant arrival cohort and assimilation effects (OLS estimates) in hospital stays and visits to emergency care (population with just NHS coverage)

	(I)	(II)	(III)	(IV)
	No. of hospitalisations		No. of visits to emergency care	
	Men	Women	Men	Women
Age effects and interactions				
Age	0.036***	0.023***	0.051***	0.029***
	(0.005)	(0.003)	(0.003)	(0.002)
$Age^2/100$	–0.068***	–0.043***	–0.112***	–0.082***
	(0.010)	(0.007)	(0.006)	(0.004)
Age^3 /10000	0.041***	0.026***	0.072***	0.058***
	(0.005)	(0.004)	(0.003)	(0.002)
Age × migrant	–0.042	–0.019	–0.020	0.046**
	(0.041)	(0.013)	(0.017)	(0.023)
Age/100 × migrant	0.112	0.026	0.043	–0.097**
	(0.111)	(0.034)	(0.039)	(0.048)
Age/10,000 × migrant	–0.082	–0.012	–0.034	0.061*
	(0.080)	(0.025)	(0.027)	(0.031)
Immigrant arrival cohort				
Pre–1996	0.255	0.247**	0.086	–0.294*
	(0.267)	(0.107)	(0.087)	(0.149)
1996–2007	0.287	0.196***	0.002	–0.187
	(0.307)	(0.071)	(0.065)	(0.125)
2008–2020	0.318	0.146***	–0.013	–0.245**
	(0.339)	(0.049)	(0.071)	(0.107)
Time of residence in Spain				
5–9 years	–0.252	–0.023	0.103**	0.022
	(0.315)	(0.027)	(0.049)	(0.085)
10–14 years	–0.299	0.036	0.156***	0.094
	(0.329)	(0.077)	(0.053)	(0.092)
15 or more years	–0.348	0.019	0.124***	0.196*
	(0.362)	(0.053)	(0.046)	(0.113)
Adjusted R^2	0.008	0.001	0.013	0.013
No. of observations	36,497	43,294	36,497	43,294
Mean of dependent variable	0.120	0.145	0.411	0.559

Notes: *** significant at 1% level; ** significant at 5% level; * significant at 10% level. All specifications include an intercept, year and region fixed effects, degree of urbanisation, education, marital and activity status and household size. Observations are not weighted. Standard errors clustered at the cohort level in parentheses.
Source: Authors' analysis from national health surveys.

Table A27. Robustness checks (V): Immigrant arrival cohort and assimilation effects (OLS estimates) in visits to GP and specialist (population aged less than 65 years)

	(I)	(II)	(III)	(IV)
	No. of visits to GP		No. of visits to specialist	
	Men	Women	Men	Women
Age effects and interactions				
Age	0.025***	0.043***	0.016***	0.022***
	(0.001)	(0.001)	(0.001)	(0.001)
$Age^2/100$	–0.036***	–0.103***	–0.023***	–0.045***
	(0.002)	(0.002)	(0.001)	(0.001)
Age^3 /10000	0.021***	0.085***	0.009***	0.031***
	(0.001)	(0.001)	(0.001)	(0.001)
Age × migrant	0.042**	0.022	0.006	–0.017
	(0.017)	(0.027)	(0.012)	(0.013)
Age/100 × migrant	–0.122**	–0.047	–0.024	0.050
	(0.049)	(0.073)	(0.033)	(0.037)
Age/10,000 × migrant	0.104**	0.030	0.022	–0.046
	(0.044)	(0.062)	(0.028)	(0.032)
Immigrant arrival cohort				
Pre–1996	–0.043	–0.200**	0.007	–0.018
	(0.050)	(0.081)	(0.036)	(0.034)
1996–2007	–0.033	–0.123*	0.009	–0.021
	(0.042)	(0.064)	(0.030)	(0.023)
2008–2020	–0.041	–0.129**	–0.018	–0.025
	(0.033)	(0.057)	(0.029)	(0.022)
Time of residence in Spain				
5–9 years	–0.013	0.065***	–0.004	–0.023
	(0.032)	(0.023)	(0.030)	(0.022)
10–14 years	0.003	0.097***	–0.014	0.023
	(0.033)	(0.030)	(0.028)	(0.030)
15 or more years	0.047	0.172***	0.024	0.027
	(0.032)	(0.041)	(0.030)	(0.032)
Adjusted R^2	0.050	0.033	0.032	0.033
No. of observations	38,245	43,327	38,245	43,327
Mean of dependent variable	0.302	0.418	0.110	0.159

Notes: *** significant at 1% level; ** significant at 5% level; * significant at 10% level. All specifications include an intercept, year and region fixed effects, degree of urbanisation, education, marital and activity status and household size. Observations are not weighted. Standard errors clustered at the cohort level in parentheses.
Source: Authors' analysis from national health surveys.

Table A28. Robustness checks (V): Immigrant arrival cohort and assimilation effects (OLS estimates) in hospital stays and visits to emergency care (population aged less than 65 years)

	(I)	(II)	(III)	(IV)
	No. of hospitalisations		No. of visits to emergency care	
	Men	Women	Men	Women
Age effects and interactions				
Age	0.039***	0.031***	0.050***	0.107***
	(0.005)	(0.003)	(0.003)	(0.003)
$Age^2/100$	–0.073***	–0.067***	–0.104***	–0.281***
	(0.008)	(0.005)	(0.005)	(0.005)
Age^3 /10000	0.045***	0.048***	0.062***	0.215***
	(0.003)	(0.003)	(0.002)	(0.003)
Age × migrant	0.057	0.055	0.026	0.076*
	(0.046)	(0.060)	(0.028)	(0.041)
Age/100 × migrant	–0.176	–0.178	–0.090	–0.193*
	(0.142)	(0.174)	(0.073)	(0.111)
Age/10,000 × migrant	0.177	0.164	0.086	0.152
	(0.148)	(0.149)	(0.061)	(0.095)
Immigrant arrival cohort				
Pre–1996	0.094	0.097	0.106	–0.389***
	(0.149)	(0.082)	(0.099)	(0.146)
1996–2007	0.136	0.073	–0.029	–0.227*
	(0.188)	(0.052)	(0.073)	(0.121)
2008–2020	0.171	0.027	–0.062	–0.301***
	(0.213)	(0.065)	(0.081)	(0.104)
Time of residence in Spain				
5–9 years	–0.225	–0.023	0.071	0.022
	(0.272)	(0.024)	(0.046)	(0.086)
10–14 years	–0.238	0.050	0.145***	0.118
	(0.283)	(0.082)	(0.052)	(0.097)
15 or more years	–0.277	0.039	0.099**	0.239**
	(0.313)	(0.051)	(0.046)	(0.111)
Adjusted R^2	0.006	0.001	0.016	0.013
No. of observations	30,295	31,240	30,295	31,240
Mean of dependent variable	0.087	0.129	0.390	0.556

Notes: *** significant at 1% level; ** significant at 5% level; * significant at 10% level. All specifications include an intercept, year and region fixed effects, degree of urbanisation, education, marital and activity status and household size. Observations are not weighted. Standard errors clustered at the cohort level in parentheses.
Source: Authors' analysis from national health surveys.

References

Zheng, B., & Agresti, A. (2000). Summarizing the predictive power of a generalized linear model. *Statistics in Medicine*, *19*(13), 1771–1781. https://doi.org/10.1002/1097-0258(20000715)19:13<1771::AID-SIM485>3.0.CO;2-P
